# Insight into the Folding and Dimerization Mechanisms of the N-Terminal Domain from Human TDP-43

**DOI:** 10.3390/ijms21176259

**Published:** 2020-08-29

**Authors:** Mirella Vivoli-Vega, Prandvera Guri, Fabrizio Chiti, Francesco Bemporad

**Affiliations:** 1 Department of Experimental and Clinical Biomedical Sciences “Mario Serio”, Section of Biochemistry, University of Florence, Viale G. B. Morgagni 50, 50134 Florence, Italy; m.vivoli2@exeter.ac.uk (M.V.-V.); prandvera.guri@stud.unifi.it (P.G.); fabrizio.chiti@unifi.it (F.C.); 2 Living Systems Institute, University of Exeter, Stocker Road, Exeter EX4 4QD, UK

**Keywords:** amyotrophic lateral sclerosis, folding intermediate, misfolding, self-assembly, neurodegeneration, proteinopathy, native-like state, chevron plot

## Abstract

TAR DNA-binding protein 43 (TDP-43) is a 414-residue long nuclear protein whose deposition into intraneuronal insoluble inclusions has been associated with the onset of amyotrophic lateral sclerosis (ALS) and other diseases. This protein is physiologically a homodimer, and dimerization occurs through the N-terminal domain (NTD), with a mechanism on which a full consensus has not yet been reached. Furthermore, it has been proposed that this domain is able to affect the formation of higher molecular weight assemblies. Here, we purified this domain and carried out an unprecedented characterization of its folding/dimerization processes in solution. Exploiting a battery of biophysical approaches, ranging from FRET to folding kinetics, we identified a head-to-tail arrangement of the monomers within the dimer. We found that folding of NTD proceeds through the formation of a number of conformational states and two parallel pathways, while a subset of molecules refold slower, due to proline isomerism. The folded state appears to be inherently prone to form high molecular weight assemblies. Taken together, our results indicate that NTD is inherently plastic and prone to populate different conformations and dimeric/multimeric states, a structural feature that may enable this domain to control the assembly state of TDP-43.

## 1. Introduction

In 2006, two seminal papers by Neumann et al. [1] and Arai et al. [2] reported that the nuclear TAR DNA-binding protein 43 (TDP-43) is involved in amyotrophic lateral sclerosis (ALS) and tau-negative, ubiquitin-positive frontotemporal lobar degeneration (FTLD-U). The authors found TDP-43 to be the main component of the characteristic intraneuronal inclusions of patients with ALS and FTLD-U. Later on, TDP-43 inclusions were also found in the brain of cases with Alzheimer’s (AD), Parkinson’s (PD) and Huntington’s (HD) diseases [3,4,5], although they are not thought to represent a primary histopathological trait in these cases.

Both sporadic ALS and FTLD-U, and a large fraction of familial cases of the same diseases, have a characteristic histopathology with polyubiquitinated, hyperphosphorylated and partially proteolyzed cytosolic inclusions of TDP-43 [1,6,7]. In ALS cases, these inclusions are found in the upper and lower motor neurons of the brain, brainstem and spinal cord, but also in the frontotemporal cortex and hippocampus in a fraction of ALS patients [1,2,8]. In FTLD-U cases, they mainly accumulate in the frontal and temporal cortices, hippocampus, with the frequent involvement of brainstem and spinal cord motor neurons [1,9].

**Figure 1:**
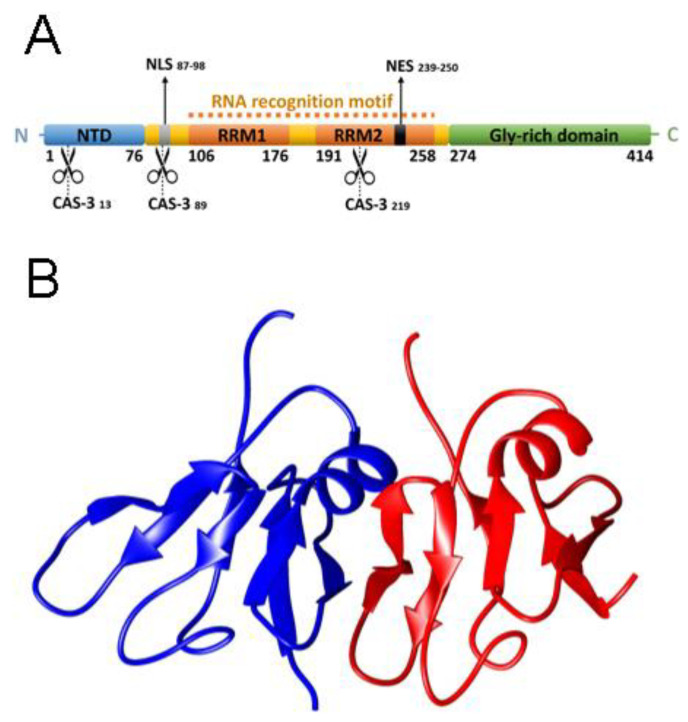
Gene architecture of TDP-43 (**A**) and structure of the NTD (**B**), spanning residues 1–80 (PDB code 6B1G) [13].

TDP-43 has 414 amino acid residues and consists of a folded N-terminal domain (NTD_1–76_) with a well-defined globular structure and shown to form a dimer or oligomer [10,11,12,13]. The protein also bears two highly conserved folded RNA recognition motifs (RRM1_106–176_ and RRM2_191–259_), required to bind sequence-specific target RNA and DNA [14,15,16,17], and an intrinsically disordered, glycine-rich C-terminal domain (CTD_274–414_) with prion-like properties [18,19,20,21] (Figure 1a). Furthermore, both a nuclear localization signal (NLS_82–98_) and a nuclear export signal (NES_239–250_) and three potential caspase-3 cleavage sites at positions 13, 89 and 219 are present [22] (Figure 1a). Different roles have been attributed to TDP-43, such as microRNA processing [23], apoptosis [24], cell division [25], mRNA stabilization, transcription, translation and splicing [26,27,28], axonal transport [28] and embryo development [29].

The NTD_1–76_ plays a very important role in the structure and oligomerization state of the full-length protein. Firstly, since it is the NTD of a long protein, it is the first to be synthesized by the ribosome and to fold [30,31,32,33]. Secondly, it dimerizes and oligomerizes in vitro when dissected from the remainder of the protein and is proposed to be responsible for the dimerization and oligomerization of the entire full-length protein [10,11,12,13]. The NTD-driven oligomerization state of TDP-43 modulates the function and propensity to aggregate the entire protein into pathological inclusions [11,12,13,30,31,34].

Starting from a low resolution structural model of the NTD backbone previously presented [32], the essentially complete ^1^H, ^1^5N and ^13^C nuclear magnetic resonance (NMR) assignments and the structure of the NTD were first reported, determined on the basis of 26 hydrogen-bonds, 60 torsion angles and 1058 unambiguous nuclear Overhauser effect (NOE) structural restraints [33]. The structure consists of a α-helix and six β-strands, with two β-strands forming a β-hairpin (PDB ID 2N4P). The fold is topologically similar to the reported structure of the C-terminal dix domain of axin 1. All X-Pro peptide populate the *trans* configuration and the two cysteine residues at positions 39 and 50 were found to be reduced and distantly separated on the surface of the protein. The protein was found to be stable and no denatured species were observed at pH 4 and 25 °C. The conformational stability of the domain, as measured by the hydrogen/deuterium exchange, was found to be 4 kcal mol^−1^. The β-strands, α-helix, and three of four turns are rigid, although the loop formed by residues 47–53 appeared mobile. Later on, the same authors reported a higher resolution NMR structure of the monomer (PDB ID 5MRG), due to a higher number of NOEs [10]. A structural model of a symmetric head-to-head dimer involving approximately residues 27–36 was also proposed using 17 cross-peaks in ^13^C-edited ^12^C-filtered 2D NOESY and following the observation that the L27A mutant adopts a monomeric state.

In an independent study, 38 hydrogen-bonds, 129 torsion angles and 829 unambiguous distance restraints were used to calculate the NMR monomeric structure (PDB ID 5X4F), which was found to have a structural fold and a secondary-structure pattern similar to the previously reported structure [12]. The authors inferred a structural model of a symmetric head-to-head dimer from the aggregation states of mutants of the NTD, leading however to a very different interface involving residues 71–74.

Another study led to a 2.1 Å X-ray structure of a non-symmetric head-to-tail dimer (PDB ID 5MDI), with a new monomer-to-monomer interface involving different residues in the two monomers [11]. Using a combination of X-ray crystallography, NMR spectroscopy and electron microscopy, the same authors proposed that the NTD adopts dynamic, solenoid-like structures, where the oligomers form from the propagation of the same head-to-tail intermolecular interface in different units. A similar head-to-tail oligomeric structure was finally presented using solution NMR (PDB ID 6B1G), except for a slight torsion of the monomer–monomer interface [13].

In this work, we studied the folding process of NTD_1–77_ (hereinafter referred to as NTD) from its fully unfolded state to its folded native dimer and identified a peculiar mechanism through which the protein domain folds with at least two parallel pathways and partially folded states accumulating in both pathways. A small subpopulation of protein molecules folds after a rate-limiting proline isomerization step. We also found that the major folding transition is followed by dimerization and that the folded state is structurally susceptible and can assemble into higher order oligomers. Using Förster resonance energy transfer (FRET), we were also able to determine the distances between cysteine residues located on different subunits in the dimer, therefore addressing which of the proposed monomer-to-monomer interfaces proposed so far is compatible with the dimeric structure in solution isolated here.

## 2. Results

### 2.1. NTD is a Folded Dimer

**Figure 2:**
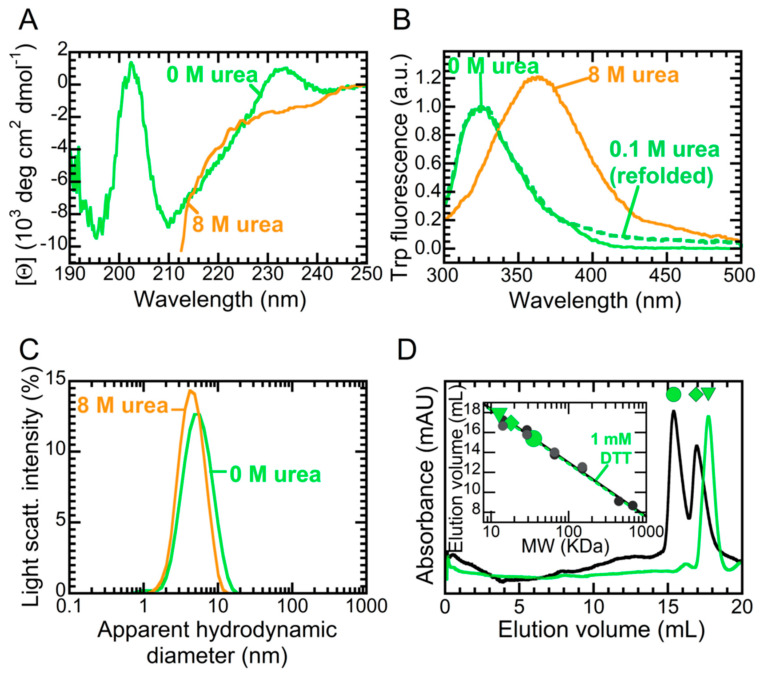
CD (**A**), tryptophan fluorescence (**B**), DLS (**C**) and analytical gel filtration analysis (**D**) of NTD in the absence (green) and presence of 8.0 M urea (orange). In panel **B**, we normalized the fluorescence signal to the maximum emission measured in the absence of the denaturant. Continuous and dashed lines refer to the folded and refolded state, respectively. In panel **D**, we reported data in the absence (black continuous line) and presence (green dashed line) of 1 mM DTT. The inset shows the calibration curves obtained with proteins of known mass in the absence (black continuous) and presence (green dashed) of 1 mM DTT.

NTD, devoid of any tag, was purified as described in the Supplementary Materials and found to be electrophoretically and chromatographically pure (>95%). As a preliminary characterization, we investigated its structure and oligomerization state under the experimental conditions selected here, i.e., 5 mM sodium phosphate buffer, pH 7.4, with 50 mM NaCl and 1 mM dithiothreitol (DTT), in the absence or presence of 8.0 M urea, 25 °C. The far-UV circular dichroism (CD) spectrum featured two negative peaks at 195 and 209 nm (Figure 2a). There was also a small positive band at 233 nm, which was absent in the spectrum measured under denaturing conditions. This spectrum was very similar to the CD spectra previously reported for other NTD constructs [12,30] and was not found to change in the presence of 150 mM NaCl (Supplementary Figure S1a), suggesting it does not depend on salt concentration. The positive band observed at 233 nm is a rather uncommon feature. Consequently, we performed the deconvolution of this spectrum using the BeStSel algorithm (http://bestsel.elte.hu), which takes into account sheet twisting as a deconvolution parameter [35]. The results of this analysis allow the band at 233 nm to be assigned to the presence of a right-hand twisted antiparallel β-sheet (Supplementary Table S1), a structural feature indeed present in all NTD sheets (Figure 1b). By contrast, the spectrum in 8.0 M urea was reminiscent of a fully unfolded state (Figure 2a).

The tryptophan fluorescence emission spectrum of NTD in the absence of denaturant revealed a single peak located at 325 nm, indicating that the indole moiety of Trp68 was well buried in the hydrophobic core (Figure 2b). In 8.0 M urea, the emission underwent a 20% increase in intensity and a red shift to ca. 355–360 nm, which is compatible with an indole fully exposed to the solvent. Furthermore, we measured fluorescence after a dilution of the unfolded protein to a final urea concentration of 0.1 M, obtaining a spectrum fully superimposable to that of the folded protein (Figure 2b). This shows that the urea-induced denaturation of NTD was a fully reversible process.

We then investigated the hydrodynamic diameter of NTD by exploiting dynamic light scattering (DLS, Figure 2c). In the absence of denaturant, the distribution of light scattering intensity vs. the apparent hydrodynamic diameter revealed a monodispersed distribution with a hydrodynamic diameter of 4.8 ± 0.3 nm. In the presence of 8.0 M urea, the hydrodynamic diameter was 4.6 ± 0.2 nm. In order to assign these experimental values to possible monomeric or multimeric states, we calculated the expected hydrodynamic diameters for different TDP-43 assemblies. Folded and unfolded monomers of TDP-43 were predicted to exhibit diameters of 3.33 and 5.20 nm, respectively, whereas an unfolded dimer would exhibit a diameter of 7.75 nm [36]. Using the PDB entry 5MDI [11], we calculated hydrodynamic diameter values of 3.23 and 4.28 nm for folded monomer and dimer, respectively. Thus, our data indicate that NTD in 0.0 M and 8.0 M urea was a folded dimer and an unfolded monomer, respectively.

Last, we characterized our NTD construct using analytical gel filtration (Figure 2d). In the absence of DTT, NTD eluted in two peaks, placed at 15.38 and 16.95 mL. Following a calibration curve obtained with standard proteins, we calculated the masses of these two peaks to be equal to 35.4 ± 1.0 and 18.1 ± 1.0 kDa, respectively. As the calculated mass of NTD was 8.53 kDa, this indicates that NTD was a dimer in equilibrium with a tetramer, under these conditions. Since other authors reported the ability of NTD to oligomerize via disulphide bridging in the absence of DTT [12], we repeated the experiment in the presence of 1 mM DTT. Under these conditions, NTD eluted as a single peak at 17.70 mL, close to that corresponding to a dimer (17.0 mL). Thus, these data indicate that the addition of DTT was sufficient to convert tetrameric NTD into a dimer. We also observed the same predominant peak in the presence of 150 mM NaCl without DTT (Supplementary Figure S1b), confirming that the dimerization state did not depend on salt concentration. In conclusion, the data presented in this section indicate that truncation of RRM1, RRM2 and CTD did not alter the secondary, tertiary and quaternary structures or the compactness of the NTD folded state. Upon addition of high amounts of urea, the folded dimer underwent a reversible transition to an unfolded monomer. The dimer appeared to be independent of salt concentration.

### 2.2. FRET Reveals That Monomers Interact Head-to-Tail in the NTD Dimer

Different groups proposed incompatible structures or structural models to describe the interactions responsible for the quaternary structure of NTD, i.e., the formation of the NTD homodimer and the structural arrangement of the monomers within the dimer. We briefly described these models in Supplementary Figure S2. In order to get insight into the structure of the NTD dimer and to assess which of the prosed structures/models is correct, we exploited FRET. Indeed, NTD has two cysteine residues, located at positions 39 and 50, which can be labeled with fluorescent probes. Thus, we produced the two variants carrying only one cysteine, namely C39S and C50S, which maintained Cys50 and Cys39, respectively. These protein variants have been already produced by another group and exhibited no altered secondary structure with respect to the wild-type protein [12]. We labeled both variants with 5-((((2-iodoacetyl)amino)ethyl)amino)naphthalene-1-sulfonic acid (1,5-IAEDANS) as a donor or 6-iodoacetamidofluorescein (6-IAF) as an acceptor. We named these fluorescent variants 39D or 39A if they carry the donor or the acceptor at position 39 (C50S mutant), 50D or 50A if they carry the donor or the acceptor at position 50 (C39S mutant). We then measured the FRET efficiency (*E*) of 1:1 molar mixtures of 39D and 39A (39D-39A), or 50D and 50A (50D-50A) in 5 mM sodium phosphate buffer, pH 7.4, 50 mM NaCl. We did not include in the analysis 1:1 mixtures of 39D and 50A (39D-50A), or 50D and 39A (50D-39A), as their measured FRET *E* values were difficult to interpret, due to multiple distances involved in each pair.

**Figure 3:**
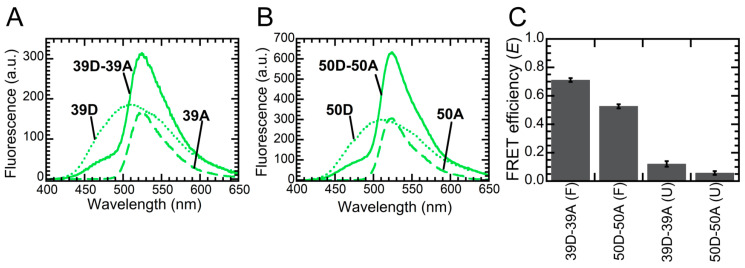
FRET measurements for NTD dimers labeled with 1,5-IAEDANS as a donor (D) and 6-IAF as an acceptor (A). (**A**,**B**) Fluorescence spectra of NTD labeled at position 39 (**A**) or 50 (**B**) with donor only (dotted line), acceptor only (dashed line) or a 1:1 molar ratio of donor and acceptor (continuous line) in the absence of urea. (**C**) FRET efficiency for NTD dimers labeled at position 39 (bars 1 and 3) or 50 (bars 2 and 4), in the absence (labeled as F) or presence (labeled as U) of 6.0 M urea. Each FRET E value is the mean of the FRET E values determined with the donor fluorescence (450–480 nm) and acceptor fluorescence (520–530 nm).

The fluorescence spectra of 39D, 39A and 39D-39A are shown in Figure 3a. The fluorescence spectra of 50D, 50A and 50D-50A are shown in Figure 3b. In both cases, emission of the donor (450–480 nm) dramatically decreased in the presence of the acceptor and emission of the acceptor (520–530 nm) increased in the presence of the donor, revealing the occurrence of FRET and thus confirming the formation of the dimer. However, the two mixtures exhibited significantly different FRET E values. The E values calculated for 39D-39A ($E_{39}$) and 50D-50A ($E_{50}$) samples were 0.72 ± 0.03 and 0.54 ± 0.03 using the decrease of donor emission, 0.70 ± 0.03 and 0.51 ± 0.03 using the increase of acceptor emission (see Methods for the procedure used). Figure 3c reports the average FRET E values for the 39D-39A and 50D-50A pairs, i.e., 0.71 ± 0.03 and 0.53 ± 0.03. Such FRET disappeared under denaturing conditions, i.e., in 6.0 M urea and 5 mM sodium phosphate buffer, pH 7.4, 50 mM NaCl, where we calculated FRET efficiencies of 0.12 ± 0.02 and 0.06 ± 0.01. This confirms that the FRET observed under native conditions arose from the intermolecular interactions that gave rise to the dimer and that unfolded NTD was a monomer (Figure 3c).

The measured FRET E values for the 39D-39A and 50D-50A pairs provide information on the spatial distances between the two residues 39 ($r_{39-39}$) and the two residues 50 ($r_{50-50}$), respectively. We used the FRET E values obtained under native conditions to calculate the $r_{39-39}/r_{50-50}$ ratio with Equation (2). This analysis yielded $r_{39-39}$/$r_{50-50}$ values of 0.88 ± 0.06, using either donor or acceptor *E* values. Among the different models taken into consideration, this value best fits with the dimer structures obtained with X-ray crystallography by Afroz et al. (5MDI), and with NMR by Wang et al. (6B1G), which give $r_{39-39}/r_{50-50}$ values of 0.67 and 0.63, respectively (Table S2). Other models imply $r_{39-39}/r_{50-50}$ values that are either below 0.5 (5MRG) or above 1.9 (5X4F). Consequently, our data support dimeric structures in which two monomers interact head-to-tail [11,13].

### 2.3. The Folded State of NTD Is Susceptible to Small Amounts of Denaturant

**Figure 4:**
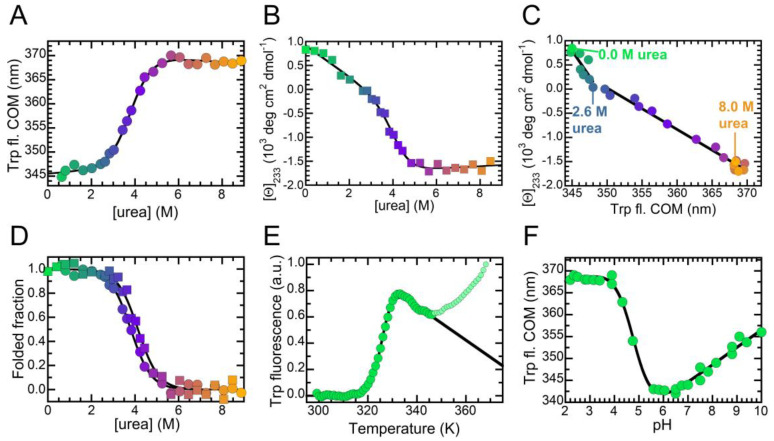
Conformational stability of NTD investigated by means of different spectroscopic probes. In all panels color ranges from green to orange as urea concentration increases from 0 to 8.4 M. (**A**) Urea-induced denaturation curve monitored by tryptophan fluorescence, reported as COM. The continuous line represents the best fit to a two-state model. (**B**) Urea-induced denaturation curve monitored by mean residue ellipticity at 233 nm. The continuous line represents the best fit to a two-state model. (**C**) Phase diagram illustrating tryptophan fluorescence (from panel **A**) plotted vs. CD signal at 233 nm (from panel **B**). Black lines highlight transitions among the conformational states detected at 0, 2.6 and 8.0 M urea. (**D**) Folded fraction as a function of urea concentration, calculated using data from panel **A** (circles) and **B** (squares). The continuous line represents the best fit to a two-state model. (**E**) Thermal denaturation monitored by means of DSF. The continuous line represents the best fit to the equation described in the Methods section. Pale green points were not included in the analysis. (**F**) pH-induced denaturation monitored by tryptophan fluorescence. The continuous line represents an arbitrary spline function.

We investigated the conformational stability of NTD by means of tryptophan fluorescence and far-UV CD (Figure 4). We prepared 28 samples containing NTD and urea concentrations ranging from 0.0 to 8.4 M and measured their tryptophan emission spectra. These spectra confirmed that NTD underwent a red shift of the fluorescence emission as the urea concentration increased (Figure S3). Thus, we calculated the center of spectral mass (COM) for each spectrum and plotted the COM value vs. urea concentration (Figure 4a). The urea-induced denaturation of NTD follows a two-state equilibrium and can be fitted to the model edited by Santoro and Bolen [37]. The results of this analysis yield values of 20.1 ± 1.5 kJ/mol, 5.3 ± 0.3 kJ/(mol M) and 3.81 ± 0.20 M for $\Delta G_{U-F}^{H_{2}O}$, $m_{\mathrm{eq}}$ and $C_{m}$, respectively. We obtained identical results in the presence of 150 mM NaCl (Figure S4). The same experiment was then repeated by following the mean residue ellipticity at 233 nm, as the difference between the signals of folded and unfolded NTD was highest at this wavelength (Figure 2a). Also in this case, the plot of the mean residue ellipticity vs. urea concentration followed a two-state model (Figure 4b). Data fitting yields values of 21.8 ± 1.5 kJ/mol, 5.3 ± 0.3 kJ/(mol M) and 4.13 ± 0.20 M for $\Delta G_{U-F}^{H_{2}O}$, $m_{\mathrm{eq}}$ and $C_{m}$, respectively.

Interestingly, the CD signal underwent a linear decrease between 0.0 and 2.6 M urea, i.e., in the pretransition region. To highlight this behavior, one can exploit a phase diagram wherein the mean residue ellipticity is plotted vs. fluorescence COM (Figure 4c). This approach illustrates two transitions. A first non-cooperative transition took place between 0.0 and 2.6 M urea and led to a distorted, yet folded, structure, with altered twisting of the β-sheet but a Trp68 indole group well buried in the hydrophobic core. The second cooperative transition took place between 2.6 and 5.4 M urea and led to a fully unfolded state, possessing a Trp68 exposed to the solvent. It is possible to follow this second transition both by CD and fluorescence, as the plots of the folded fraction calculated with either technique were superimposable (Figure 4d). The small shift of the $C_{m}$ value from 3.81 ± 0.20 M to 4.13 ± 0.20 M was indicative of the additional non-cooperative transition monitored with CD that leads to overestimate the $C_{m}$ value with this optical probe.

Next, we measured the thermal stability of our NTD construct exploiting differential scanning fluorimetry (DSF, Figure 4e). Upon increasing the temperature, we observed that Sypro orange fluorescence remained unaffected until 317 K (44 °C); above this temperature, a thermal transition occurred, which was complete at 335 K (62 °C). The signal then decreased due to aggregation and a second transition was visible above 345 K (72 °C), possibly corresponding to the disassembly of the aggregates. We fitted the data of the first region of the plot to a two-state thermal transition and obtained a melting temperature ($T_{m}$) of 326 ± 1 K (53 ± 1 °C). We also investigated the acid-induced denaturation, monitoring tryptophan emission as a function of pH (Figure 4f). The COM of the folded state appeared to depend linearly on pH, varying from 343 to 355 nm as the pH increased from 6.0 to 10.0. This suggests a structurally plastic folded state that undergoes slight modifications due to the pKa values of surface side chains. However, a clear unfolding transition, leading to an acid unfolded state, was evident between pH values of 6.0 and 4.0. Below pH 4.0, NTD exhibited no further conformational changes.

As a further control, we measured the conformational stabilities of our labeled NTD variants and of the 1:1 molar mixtures used for FRET measurements: 39D, 39A, 39D-39A, 50D, 50A and 50D-50A. The experimental data are shown in Supplementary Figure S5 and Supplementary Figure S6. The results of the fitting procedure are summarized in Supplementary Table S3. All the labeled variants were folded and showed a two-state transition. They were all less stable than the wild-type protein, with $\Delta G_{U-F}^{H_{2}O}$ values ranging from 13.9 ± 1.5 (39D) to 17.3 ± 1.5 kJ/mol (50A). 

### 2.4. Folding Kinetics of NTD Reveal Four Exponential Phases and a High Energy Intermediate State

**Figure 5:**
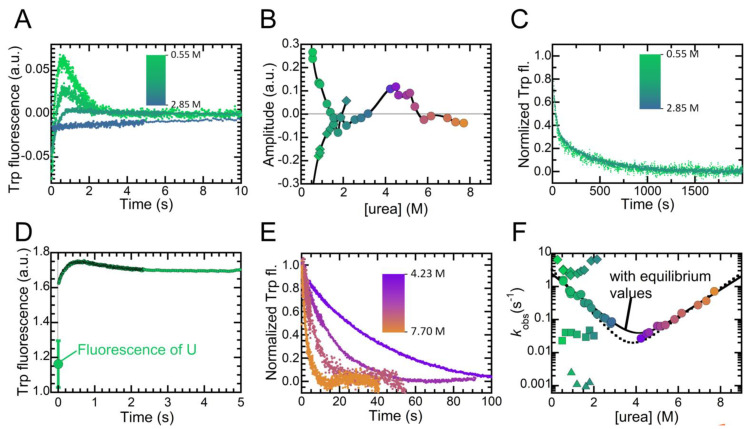
Folding kinetics of NTD. (**A**) Refolding traces monitored by tryptophan fluorescence at the urea concentrations indicated. (**B**) Amplitude of the first (diamonds) and second (circles) folding exponential phases, and of the unique unfolding phase (circles) as gauged by best fits of experimental kinetic traces to double exponential equations, plotted as a function of denaturant concentration. Color scale as in panel **A** and **C**. (**C**) Third and fourth refolding phase of NTD, monitored by tryptophan fluorescence. Color scale as in panel **A**. (**D**) Comparison between a refolding trace measured in 0.6 M urea and the fluorescence intensity of the unfolded state (U) under the same conditions, determined from a linear extrapolation of the signals emitted at high urea concentrations. (**E**) Unfolding traces monitored by tryptophan fluorescence recorded at the indicated urea concentrations. We normalized these traces to the fraction of folded protein. (**F**) Folding/unfolding rate constants of NTD plotted vs. urea concentration. Data are shown for the first (diamonds, associated with $k_{1}$), second (circles, associated with $k_{2}$ and $k_{u}$), third (squares, associated with $k_{3}$) and fourth (triangles, associated with $k_{4}$) observed phases. Color scale as in panels **A** and **E**. Dashed and continuous lines correspond to the best fits of the experimental data to Equations (3) and (4), respectively.

We investigated the folding/unfolding of NTD in real-time, monitoring intrinsic fluorescence during the process with a stopped-flow device (Figure 5). The refolding reaction was initiated by a ten-fold dilution of the urea-unfolded protein into refolding buffer containing lower urea concentrations, to final values ranging from 0.27 to 2.85 M. At very low denaturant concentration, refolding of NTD occurred in two phases: the first phase involved an increase in fluorescence and was complete in ca. 1 s; the second phase involved a decrease of fluorescence and was complete in ca. 6 s (Figure 5a). We indicated the rate constants of these two phases as $k_{1}$ and $k_{2}$, respectively. The amplitudes of the observed phases varied with urea concentration, becoming smaller as the denaturant concentration increased, and inverting their sign above 1.8 M urea (Figure 5b). We also followed the refolding of NTD on a longer time-scale, using a conventional fluorimeter rather than the stopped-flow apparatus. This experiment revealed two more apparent refolding phases (Figure 5c), whose rate constants will be indicated as $k_{3}$ and $k_{4}$, respectively. Both phases involved a decrease in tryptophan fluorescence emission, and last ca. 100 and 3000 s, respectively. The amplitudes of these two phases did not vary with urea concentration. Next, we compared the fluorescence emitted by the unfolded state under conditions that promote NTD refolding with the fluorescence observed at the beginning of a refolding experiment performed under identical conditions (Figure 5d). This experiment was conducted in 0.55 M urea. These fluorescence values were found to be 1.16 ± 0.13 a.u. and 1.61 ± 0.03 a.u., respectively. This latter value was significantly higher than that of the unfolded protein, demonstrating that the conformational state populated by NTD at the beginning of the refolding experiments was a collapsed non-folded state, characterized by a partially buried tryptophan residue. The formation of such collapsed state escaped direct experimental detection because the process was fast and occurs within the dead time of our stopped-flow kinetics, that was 6.1 ms. We will refer to this ultrafast immeasurable rate constant as $k_{U\to \mathrm{CS}}$.

Unfolding of the protein was induced by a ten-fold dilution of the folded protein into unfolding buffers containing high urea concentrations, to reach final values ranging from 4.2 to 7.7 M. Unfolding at 4.2 M followed a single exponential phase involving a decrease in fluorescence and lasted ca. 100 s (Figure 5e). As the denaturant concentration increased, the process became increasingly faster, while its amplitude decreased and became negative above 6.0 M urea (Figure 5c). The rate constant of this unfolding phase will be referred to as $k_{u}$.

We collected the observed kinetic constants ($k_{\mathrm{obs}}$) for the different folding/unfolding phases and plotted them vs. the denaturant concentration, to obtain the so-called chevron plot (Figure 5f). $k_{1}$ exhibited a peculiar trend, as its values decreased from 0.0 to ca. 0.8 M urea, remained constant from ca. 0.8 to ca. 1.2 M urea and then increased upon increasing the denaturant concentration further. This trend resembled that of an independent chevron plot (Figure 5f). Consequently, the values obtained can be fitted to Equation (3), giving fit output values of 14.9 ± 1.5 and 0.62 ± 0.06 s^−1^ for $k_{1}^{H_{2}O}$ and $k_{-1}^{H_{2}O},$ i.e., the direct and reverse constants in the absence of denaturant, respectively. The dependences of these rate constants on urea concentration ($m_{1}$ and $m_{-1}$) were 8800 ± 900 and 2800 ± 900 J/(mol M). This led to values of 7800 ± 700 J/mol, 11600 ± 1200 J/(mol M) and 0.68 ± 0.07 M for the $\Delta G^{H_{2}O},$ m value and $C_{m}$ of this hypothetical transition, respectively. 

We then analyzed the chevron plot obtained by joining the plots of $k_{2}$ and $k_{u}$. This plot shows two apparently linear limbs, with no downward curvatures at very low or very high urea concentrations. We thus checked whether this plot describes the transition between the unfolded and folded states observed at equilibrium. Fitting the experimental data to Equation (3) yielded values of 3.28 ± 0.30 s^−1^, (2.16 ± 0.20) · 10^−4^ s^−1^, 3880 ± 400 J/(mol M) and 2580 ± 300 J/(mol M) for $k_{2}^{H_{2}O}$, $k_{u}^{H_{2}O}$, $m_{2}$ and $m_{u}$, respectively. These values could be used in turn to calculate $\Delta G_{U-F}^{H_{2}O}$, $m_{\mathrm{eq}}$ and $C_{m}$ as 23900 ± 3100 J/mol, 6460 ± 1000 J/(mol M) and 3.7 ± 0.2 M, respectively. These values were, within experimental error, those reported above at equilibrium. We also fitted the experimental data to Equation (4), imposing the $\Delta G_{U-F}^{H_{2}O}$ and $m_{\mathrm{eq}}$ values obtained at equilibrium. The output of this procedure prompted us with values of 2.46 ± 0.6 s^−1^ and 2970 ± 800 J/(mol M) for $k_{2}^{H_{2}O}$ and $m_{2},$ respectively. These values are within experimental error similar to these obtained with Equation (3), indicating the transient formation of an intermediate, which possesses buried tryptophan side chains but is highly unstable, having the same energy as the unfolded state. As far as $k_{3}$ and $k_{4}$ are concerned, these two rate constants did not show clear dependencies on urea concentration. The average values of these constants were (3.55 ± 0.31) · 10^−2^ and (1.55 ± 0.38) · 10^−3^ s^−1^, respectively.

### 2.5. Folding of NTD Proceeds through a Hydrophobic Collapse Followed First by Structural Rearrangements, then by Dimerization

**Figure 6:**
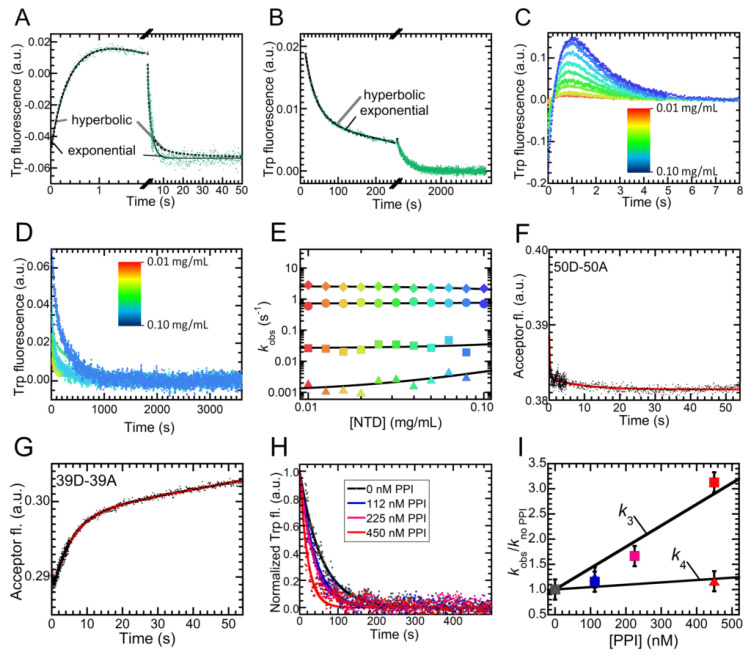
Insight into the NTD refolding process. (**A**,**B**) Comparison between the best fits to a hyperbolic (dashed line) and an exponential (continuous line) equation of one refolding trace measured in 1.2 M urea (**A**) and in 1.6 M urea (**B**). Time scales are suitable to observe the first and second (**A**) or the third and fourth (**B**) phases. In both panels, axis breaks separate the two investigated phases. (**C,D**) Analysis of NTD refolding as a function of protein concentration. Data are shown for the first and second (**C**) or the third and fourth (**D**) refolding phases. In both panels color ranges from red to blue upon NTD concentrations increasing from 0.01 to 0.1 mg/mL (1.16–11.6 µM). (**E**) Refolding rate constants as a function of NTD concentration for the first (diamonds), second (circles), third (squares) and fourth (triangles) refolding phase, as determined by best fits of experimental data to double exponential equations. (**F**,**G**) Fluorescence of the 6-IAF acceptor monitored during refolding of a 1:1 molar ratio of NTD labeled with a donor and acceptor at position 50 (50D-50A, panel **F**) and 39 (39D-39A, panel **G**). Continuous lines represent best fits of experimental data to a double exponential equation. (**H**) Third and fourth phases of refolding recorded at different PPI concentrations, ranging from 0.00 to 0.45 μM. Continuous lines represent best fits of experimental data to double exponential equations. (**I**) Ratio between the refolding rate constant measured at a given PPI concentration ($k_{\mathrm{obs}}$) for the third and fourth refolding phase and the value measured in the absence of PPI ($k_{\mathrm{no}\;\mathrm{PPI}}$ ). Colors as in panel **H**.

Given the complexity encountered in NTD folding and the high number of experimental folding phases, we set out to perform a series of experiments with the goal of assigning these phases to specific microscopic processes (Figure 6). First, we investigated whether any of the four refolding phases can be assigned to an intermolecular process—be it dimerization of folded NTD or aggregation—rather than to intramolecular conformational changes. We exploited the fact that the equations describing the time-course of dimerization and folding contain hyperbolic and exponential functions, respectively. Consequently, we checked whether fitting any of the four refolding phases of a representative refolding trace (1.2 M and 1.6 M urea) to a hyperbolic function significantly improved fitting parameter errors. In all cases, fitting did not improve by exploiting hyperbolic functions (Figure 6a,b). We also investigated all four refolding phases as a function of protein concentration, based on the rationale that any intermolecular process would necessarily become faster as protein concentration increases, unlike intramolecular conformational changes. We recorded folding kinetics at NTD concentrations ranging from 0.01 to 0.1 mg/mL (1.16–11.6 µM) in 0.83 M urea on the various time scales (Figure 6c,d). The trend of the four rate constants vs. the NTD concentration is shown in Figure 6e. This graph shows that the first three constants did not correspond to any intermolecular process, because the *r* scores for linear fits of $k_{1}$, $k_{2}$ and $k_{3}$ were 0.378, 0.198 and 0.230 (*p* > 0.05). The only significant correlation was found in the case of $k_{4}$, with an r score of 0.745 (*p* = 0.013). A DLS distribution obtained at the end of the fourth observed phase (but not before) highlighted the presence of aggregates, with apparent hydrodynamic diameters higher than 100 nm (not shown). This allows this fourth phase to be assigned to sample aggregation and precipitation, and to be excluded as a true folding phase. Moreover, the rate constant did not increase by 10 times over the investigated 10-fold protein concentration range, ruling out that this phase arises from a pure dimerization step.

The question remains as to which one, among the phases observed, corresponds to NTD dimerization. In order to answer this question, we exploited FRET. In particular, we studied folding of NTD labeled with donor and acceptor at position 50 (50D-50A, Figure 6f) and 39 (39D-39A, Figure 6g) in 0.45 M urea. We monitored the occurrence of dimerization following in real-time the fluorescence emitted by the acceptor, using a band-pass filter cutting out fluorescence below 470 nm. In the case of 50D-50A, the signal during refolding underwent a small modification, probably because the decrease of donor fluorescence and the increase of acceptor fluorescence cancel each other following dimerization. Acceptor fluorescence decreased from 0.386 to 0.382 a.u. (Figure 6g). However, in the case of 39D-39A, we observed that the fluorescence emitted by the acceptor increased from 0.288 to 0.302 a.u. in ca. 50 s, following a biexponential trend. The rate constant of the second phase ($k_{\mathrm{DIM}}$), which involves the increase in signal, determined from best fits of experimental data of Figure 6g to a double exponential equation, appeared to be 0.30 ± 0.03 s^−1^. This value was significantly smaller than the $k_{2}$ value measured with intrinsic fluorescence under the same conditions (1.40 ± 0.14 s^−1^). Consequently, this experiment indicates that dimerization of NTD could not be observed by means of intrinsic tryptophan fluorescence. The process occurred only after refolding was compete.

The third refolding phase took place on a time-scale compatible with proline isomerization. The primary sequence of NTD bears five proline residues, all in a *trans* configuration in the folded state. Thus, it is possible that a subpopulation of protein molecules refolds slower, in a process rate-limited by the isomerization of one or more specific X-Pro peptide bond(s). To test this hypothesis, we followed the third and fourth observed phases in the absence or presence of increasing amounts of a peptidyl-prolyl-isomerase (PPI), namely macrophage infectivity potentiator (Mip) (Figure 6h). The results show that the third phase becomes faster as the PPI concentration increases. The $k_{3}$ values were (2.21 ± 0.20) · 10^−2^ and (6.90 ± 0.50) · 10^−2^ s^−1^ in the absence and presence of 450 nM PPI (Figure 6I). Such an increase could not be observed for $k_{4}$. Consequently, the third phase could be assigned to proline isomerism: a small subpopulation of protein molecules cannot refold until one or more specific X-Pro peptide bond(s), in the wrong *cis* configuration, undergo isomerization to the *trans* configuration.

## 3. Discussion

### 3.1. A Model for the Folding and Dimerization of NTD

The prion-like CTD seems to underlie the aggregation propensity of TDP-43 and most mutations linked to familial ALS (fALS) are located within this domain [38,39]. However, scattered reports indicate that the NTD may play a pivotal role in regulating the assembly state of TDP-43. Single-molecule measurements coupled to bulk biophysical techniques indicated that TDP-43 converts into amyloid-like fibrils following two distinct pathways. In one pathway, the CTDs of different molecules assemble directly [34]. In a second, parallel, pathway, fibrillation is induced by the NTD following a two-step process in which first the NTD domains dock, then the CTDs lock the molecules into an amyloid-like aggregate [34]. This is consistent with a previous finding that the first 10 residues of the TDP-43 sequence are crucial for both the folding and misfolding of the protein [31]. Indeed, by removing such a segment, TDP-43 appears to no longer be able to aggregate in the cytosol, even if the cytoplasmic accumulation of the protein is enhanced by removing the NLS sequence. However, other authors suggested that the NTD-driven homo-polymerization might exert a protective role against pathological aggregation. For example, it was reported that NTD dimerization or tetramerization via disulfide bridges may prevent aggregation of TDP-43 and is necessary for the splicing activity of the protein [12]. Other authors reported that TDP-43 oligomerizes in the nucleus via the NTD [11]. This may separate spatially the CTDs of different TDP-43 molecules, therefore antagonizing pathological aggregation. Alterations of this equilibrium lead to the accumulation of TDP-43 in the cytoplasm, where the protein aggregates irreversibly, via the CTD [11].

**Figure 7:**
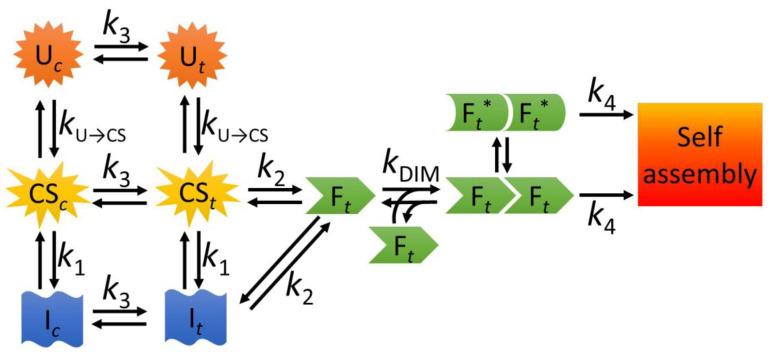
A model describing the folding, unfolding, dimerization and possible misfolding equilibria of NTD. U, unfolded state; CS, collapsed state; I, intermediate state; F, folded state. The subscripts *t* and *c* indicate the *trans* and *cis* configurations of one or more specific X-Pro peptide bond(s). F_t_*, a native-like state with distorted β-sheet structure.

Be it protective or detrimental, the NTD-controlled multimerization appears to play a major role in the aggregation properties of TDP-43 and, consequently, the identification of possible alternative conformational ensembles populated either transiently or permanently by this domain can help understand the behavior of the bulk protein. Based upon the entire body of experimental evidence presented in this manuscript, we can build a model underpinning the different steps of the folding pathway of NTD (Figure 7). This model is able to describe a series of conformational states populated transiently by NTD during its folding. The unfolded state exists in a pre-equilibrium between molecules having all the proline residues in *trans* (U*_t_*) and a subset of molecules, possessing one or more specific X-Pro peptide bonds in the *cis* configuration (U*_c_*). Both these unfolded states convert, within the dead time of our stopped-flow experiments (6.1 ms), into an ensemble of collapsed states, which again can possess all proline residues in *trans* (CS*_t_*), or one or more X-Pro peptide bonds in *cis* (CS*_c_*). This process is associated with the $k_{U\to \mathrm{CS}}$ rate constant. We surmised this fast step from the difference between the fluorescence emission observed at the beginning of the refolding process and that of the unfolded state. Albeit not yet folded, the CS state possesses a buried indole moiety of Trp68. The CS can then convert into the folded state following two parallel pathways. In the first pathway, CS converts into an intermediate state (I), in a process associated with the $k_{1}$ rate constant. This I state, again, can have all prolines in *trans* (I*_t_*) or one or more X-Pro peptide bond in *cis* (I*_c_*). The I*_t_* state is on the pathway of folding and can convert into the folded state, in a process associated with the $k_{2}$ rate constant. The on-pathway nature of the I state was supported by the observation that $k_{-1}^{H_{2}O}$ was 0.62 ± 0.06 s^−1^, a value smaller than $k_{2}^{H_{2}O}$, equal to 3.28 ± 0.30 s^−1^. This is compatible only with an on-pathway model [40]. At very low urea concentrations, the I state is relatively stable and possesses energy similar to that of the CS ensemble. This is shown by the fact that the chevron plot for $k_{2}$ and $k_{u}$ can be analyzed with a two-state model and does not exhibit downward curvatures at low urea concentration (Figure 5f). As urea concentration increases, I becomes more unstable than CS and this leads to a second, parallel, folding pathway, in which CS*_t_* undergoes a direct transition that leads to the formation of the folded state (F), with no need of intermediate states accumulating during the process. The occurrence of this pathway was demonstrated by the trend observed in Figure 5f between denaturant concentrations of 0.67 and 3.70 M, i.e., between the two $C_{m}$ values for the chevron plots of $k_{1}$ and $k_{2}$ (with $k_{u}$). Within this range of concentrations, $k_{-1}$ was increasing upon increasing urea concentration, indicating denaturation of the I state, but $k_{2}$ was instead detectable, because folding was still favorable. This means that, under these conditions, NTD underwent folding even though I was less stable than CS and, therefore, folding proceeded directly from CS*_t_* to F*_t_*, while I was no longer accumulated. It is also possible that I still forms under these conditions (CS*_t_* → I*_t_* →F*_t_*), in a folding process where it has an energy value intermediate between that of CS*_t_* and the transition state for folding, therefore remaining undetected.

In either pathway (CS*_t_* →F*_t_* or I*_t_* →F*_t_*), formation of the folded state can occur only if all X-Pro peptide bonds are in the correct *trans* configuration. We observed that a small subset of protein molecules, possessing one or more specific X-Pro peptide bond(s) in the wrong *cis* configuration, fold only after proline isomerism, associated with the $k_{3}$ rate constant. Last, the F state is able to dimerize, with a rate constant ($k_{\mathrm{DIM}}$) significantly lower than that of refolding ($k_{2}$), indicating that NTD dimerization follows its folding. Dimerization gives rise to a head-to-tail homodimer (F*_t_*F*_t_*), as assessed by our FRET measurements. The head-to-tail arrangement we identified for the dimer is not only in agreement with other models previously reported [11,13] but it is also compatible with the idea of a NTD-driven polymerization of TDP-43. Indeed, a head-to-tail dimer is inherently prone to further polymerization [11], whereas a head-to-head (or tail-to-tail) dimer has no docking sites free and exposed to the solvent and needs to undergo at least one further misfolding step prior to becoming prone to aggregation. Indeed, we found that the folded state of NTD is able to self-assemble into higher molecular weight assemblies ($k_{4}$).

Of note, the folded dimer is prone to undergo structural rearrangements and populate a native-like state (F_t_*), as confirmed by our finding that small amounts of urea can distort the twisting of the β-sheets without inducing full denaturation and that small modifications to the pH alter the chemical environment surrounding the tryptophan side chain. Interestingly, the F state of NTD can form tetramers via disulphide bridging, as found in our experiments and by other authors [12]. When isolated via gel filtration, the tetramer appeared less stable than the dimer, as we found $T_{m}$ values of 47.2 °C (Figure S7) and 52.8 °C for tetramer and dimer, respectively. While it is presently difficult to explain the destabilization observed for the tetramer, these findings lend further support to the idea of a structurally susceptible folded state of NTD, whose oligomerization may trigger further assembly.

### 3.2. The Role of Partially Folded Conformations in Misfolding

The identification of conformational ensembles other than the fully folded state and populated transiently or permanently can be crucial to understand the factors that alter the subtle equilibrium between folding and misfolding. Several cases there exist in the literature of partially folded states able to give rise to aggregation. For example, NMR experiments carried out on a triple mutant of the Fyn Src Homology 3 (SH3) domain revealed that an intermediate state formed during folding bears a disordered C-terminal segment. The displacement of the C-terminus exposes a normally buried β-sheet, an event that in turn makes the protein prone to self-assemble [41]. A series of mutants of the SH3 domain from α-spectrin possess an unfolded β-strand, which leads the protein to self-assemble [42]. In this case, the same residues that are fundamental in the folding process also trigger aggregation. Other authors identified aggregation prone intermediates in the mouse prion protein [43], in the superoxide dismutase [44] and in the neuronal calcium sensor-1 [45], thus suggesting that the formation of partially folded states prone to aggregate is more than a rare occurrence. In our group, we recently identified a partially folded state (PF) populated on the folding pathway of human profilin-1 and its pathological mutants associated with the onset of fALS [46]. This PF state is prone to aggregate, as shown by its ability to interact with preformed aggregates, increasing the signal of the amyloid-reporting dye Thioflavin-T [47]. Furthermore, the PF becomes more stable in those mutants that are disease-involved and prone to aggregate, allowing a correlation between PF stability and aggregation propensity to be established in this group of mutants [47].

An equilibrium folding intermediate was identified even in the RRM2 domain of TDP-43 [48]. This may play a physiological role, as it is believed to enhance the access to the NES sequence of TDP-43. The same conformational state may also play an important role in promoting TDP-43 self-assembly and the interactions between RRM1 and RRM2 reduce the population of the intermediate, increasing the binding to nucleic acids. Intriguingly, the interaction with nucleic acids was documented even for NTD [32], and this raises the question as to whether nucleic acids may be able to alter the relative stabilities of the conformational ensembles populated by NTD and, consequently, its propensity to undergo self-assembly.

Interestingly, even alterations of proline isomerism equilibria have been previously linked to misfolding. In the case of β2-microglobulin (β2m), a native-like partially folded state was described, which possesses one specific X-Pro peptide bond in the non-native *trans* configuration. The formation of this state leads to an increase in structural flexibility and fluctuations [49] and this conformation is 5 times more prone to aggregate, when compared to the fully folded state [50]. Accordingly, NMR experiments showed that when the population of this ensemble decreases, aggregation of β2m becomes less favorable as the energy barrier for misfolding increases [51]. Thus, our identification of an X-Pro isomerism event during NTD folding may deserve further investigation with respect to the bulk behavior of the protein.

### 3.3. NTD as a Highly Plastic Protein Domain

We identified herein a number of conformational states distinct from the fully folded and unfolded states and accumulating during the refolding of NTD. In particular, we characterized a collapsed state forming immediately at the initiation of folding (CS) and an intermediate state forming on the timescale of a few milliseconds and in equilibrium with the CS (I). Furthermore, we identified conformational states having one or more X-Pro peptide bonds in an incorrect cis configuration (CS*_c_* and I*_c_*), a monomeric folded state forming before dimerization (F*_t_*) and a native-like dimeric state in equilibrium with the folded dimer (F_t_*F_t_*). While in the case of NTD it is presently difficult to assess the real propensity to self-assemble of the various conformational states identified, a general picture is emerging in which any increase in structural flexibility, together with the ability to populate partially distorted conformations, may trigger self-assembly. Our characterization of the folding pathway of NTD represents a first step towards the full description of the TDP-43 landscape. Further studies will get further insight into the relative propensities to self-interact of the different conformational ensembles identified in the present study.

## 4. Materials and Methods 

### 4.1. Gene Cloning, Mutagenesis, Expression and Purification

We reported the protocols for gene cloning, mutagenesis, expression and purification in the Supplementary Materials.

### 4.2. Far-UV Circular Dichroism Spectroscopy

We recorded far-UV CD spectra of NTD at a concentration of 0.2 mg/mL (23.5 µM) in 5 mM sodium phosphate buffer, 50 mM NaCl and 1 mM DTT, pH 7.4, in the absence and presence of 8.0 M urea. We used a 0.5 mm cuvette from Hellma and collected data from 190 to 250 nm at 25 °C using a J-810 Spectropolarimeter from Jasco (Tokyo, Japan) equipped with a thermostated cell holder attached to a Thermo Haake C25P water bath (Karlsruhe, Germany). We subtracted spectra of blanks containing only buffers, masked the data when the high tension (HT) signal was >600 V and converted them to mean residue ellipticity ([Θ]).

### 4.3. Fluorescence Spectroscopy

We prepared two samples, containing 0.04 mg/mL (4.7 µM) NTD in 5 mM sodium phosphate buffer, 50 mM NaCl and 1 mM DTT, pH 7.4, in the absence and presence of 8.0 M urea. We then acquired fluorescence spectra using a 10 mm × 4 mm quartz cuvette. We collected data between 300 and 500 nm at 25 °C, with an excitation wavelength of 280 nm, using a PerkinElmer LS 55 spectrofluorimeter (Waltham, MA, USA), equipped with a thermostated cell holder attached to a Haake F8 water bath (Karlsruhe, Germany). Excitation and emission slits were 5 nm. In a second experiment, we prepared a sample containing 1.20 mg/mL (141 µM) NTD in 5 mM sodium phosphate buffer, 50 mM NaCl, 1 mM DTT, pH 7.4, containing 6.0 M urea. This sample was incubated for 1 h and then diluted 60 times with the same buffer without urea, to reach final conditions of 0.02 mg/mL (2.35 µM) NTD in 5 mM phosphate buffer, 50 mM NaCl, 1 mM DTT, pH 7.4 and 0.1 M urea. We then acquired the fluorescence spectrum using the same cuvette and instrumental settings, normalized this spectrum to a protein concentration of 0.04 mg/mL (4.7 µM) and plotted it with the spectrum of the folded protein.

### 4.4. Dynamic Light Scattering

We prepared two samples containing 0.5 mg/mL (58.7 µM) NTD in 5 mM sodium phosphate buffer, 50 mM NaCl and 1 mM DTT, pH 7.4, and 0.4 mg/mL (47 µM) NTD in 5 mM sodium phosphate buffer, 50 mM NaCl, 1 mM DTT and 8.0 M urea, pH 7.4. Samples were centrifuged at 24,000× *g* for 15 min, at 4 °C, and filtered with Whatman Anotop 0.02 µm cut-off filters, in order to remove aggregates. We then acquired their size distributions (distribution of apparent hydrodynamic diameter by light scattering intensity) at 25 °C, using a Malvern Panalytical Zetasizer Nano S DLS device (Malvern, Worcestershire, UK). We used a 10 mm plastic cuvette, considering the refractive index and viscosity of the buffer solutions as 1.333 and 0.87 cp (first sample) and 1.40 and 1.44 cp (second sample), respectively.

### 4.5. Analytical Gel Filtration

We carried out gel filtration using a Superdex^®^ 200 Increase 10/300 GL (GE Healthcare, Chicago, IL, USA) pre-equilibrated at 4 °C with 5 mM phosphate buffer, 50 mM NaCl, pH 7.4. We performed the experiment in the presence or absence of 1 mM DTT. 100 µL of NTD were loaded at a concentration of 0.5 mg /mL (58.7 µM) in 5 mM sodium phosphate buffer, 50 mM NaCl, pH 7.4. We determined a calibration curve to interpolate molecular weights using elution volumes, as previously reported [52]. Briefly, 100 µL of the following standards were loaded separately: thyroglobulin (669 kDa), apoferritin (443 kDa), alcohol dehydrogenase (150 kDa), albumin (66 kDa), carbonic anhydrase (29 kDa) and α-lactalbumin (14 kDa). These samples were used to make a calibration curve using their known molecular weights and their experimental elution volumes. Using these data, we interpolated the molecular weights and, thus, the oligomeric states of NTD samples.

### 4.6. Labelling

We exploited the thiol-reactive probes (1,5-IAEDANS (donor) and 6-IAF (acceptor) to label the single cysteine residues present in the C39S and C50S mutants of NTD. Both fluorescent probes (Thermo Fisher Scientific, Waltham, MA, USA) were separately dissolved in dimethylformamide (DMF) at 60 mM concentration. Of each protein sample 5 µL were added to 500 µL in 5 mM sodium phosphate buffer and 50 mM NaCl, pH 7.4, whose protein concentration was 0.5 mg/mL (58 µM) and incubated for 16 h at 20 °C with gentle and constant shaking. In these samples the probe:protein molar ratio was 10:1, i.e., 594:58 µM. This was carried out for both mutants and for both probes for a total of four samples (39D, 39A, 50D and 50A). After 16 h, the excess of unreacted probe was removed by gravity chromatography using 6 mL of G-15 resin (Pharmacia, Uppsala, Sweden), previously equilibrated with 5 mM sodium phosphate buffer and 50 mM NaCl, pH 7.4, at 25 °C. Labeled protein fractions were collected and pooled according to their absorbance values at 280 nm and 336 nm, then concentrated using 5 centrifugal filter devices with a 3 kDa molecular weight cut off (MWCO) cellulose membrane (Millipore, Burlington, MA, USA) and stored at −20 °C before use.

### 4.7. Förster Resonance Energy Transfer (FRET)

We mixed at a 1:1 molar ratio the C39S (or C50S) mutant labeled with 1,5-IAEDANS (50D or 39D) with the C39S (or C50S) mutant labeled with 6-IAF (50A or 39A), to a total protein concentration of 0.1 mg/mL (11.7 µM). Experimental conditions were 5 mM sodium phosphate buffer and 50 mM NaCl, pH 7.4, 25 °C, in the absence or presence of 6.0 M urea. This led to two different combinations of donor and acceptor: 39D-39A and 50D-50A. Four samples containing only 50D, 50A, 39D and 39A at the corresponding probe concentration were also prepared, to a total protein concentration of 0.05 mg /mL (5.9 µM). We acquired the fluorescence spectra of the various samples, using a 10 mm × 2 mm quartz cuvette, between 400 and 650 nm at 25 °C, with an excitation wavelength of 336 nm (donor excitation wavelength), using a PerkinElmer LS 55 spectrofluorimeter (Waltham, MA, USA) equipped with a thermostated cell holder attached to a Haake F8 water bath (Karlsruhe, Germany). The fluorescence spectrum of the 39D-39A sample was cleaned from the contributions of the 39D-39D and 39A-39A populations, as these do not produce FRET. This was achieved using the equation $F_{39D-39A}=F_{39D-39A}^{\prime }-0.5\cdot \left(F_{39D}^{\prime }+F_{50D}^{\prime }\right)$, where F is the fluorescence emission of the sample indicated by the subscript and the prime denotes the uncorrected signal.

We then calculated the FRET efficiency (E) using either the decrease in donor emission, or the increase in acceptor emission. In the case of the donor, E at position 39 ($E_{39}$) and 50 ($E_{50}$) were calculated as $E_{39}=1-\left(F_{39D-39A}/F_{39D}\right)$ and $E_{50}=1-\left(F_{50D-50A}/F_{50D}\right)$. In the case of the acceptor, we calculated $E_{39}$ and $E_{50}$ as $E_{39}=\left[\left(F_{39D-39A}-F_{39A}\right)/F_{39A}\right]\cdot \left(A_{39A}/A_{39D}\right)$ and $E_{50}=\left[\left(F_{50D-50A}-F_{50A}\right)/F_{50A}\right]\cdot \left(A_{50A}/A_{50D}\right)$, where A was the absorption at 336 nm of the sample indicated by the subscript. For the signal of the donor, we used the band between 450 and 480 nm, where the acceptor did not emit. For the signal of the acceptor, we exploited the band between 520 and 530 nm. In this latter case data were affected by the fact that both D and A emit in the range 520–530 nm. Consequently, the contribution of the donor was removed by subtracting the donor spectrum ($F_{39D}$ or $F_{50D}$), normalized to the donor emission at 450–480 nm observed in $F_{39D-39A}$ or $F_{50D-50A}$, from the spectrum of the donor-acceptor mix ($F_{39D-39A}$ or $F_{50D-50A}$).

One can easily convert *E* into an inter-residue distance r, using:

(1) $$r=R_{0}\cdot \sqrt[{6}]{\frac{1}{E}-1},$$

where *R*_0_ is the Förster distance. The ratio of the spatial distance *r* between residues 39 ($r_{39-39}$) and residues 50 ($r_{50-50}$) in the dimer was obtained from Equation (1) as:

(2) $$\frac{r_{39-39}}{r_{50-50}}=\sqrt[{6}]{\frac{\left(1-E_{39}\right)\cdot E_{50}}{\left(1-E_{50}\right)\cdot E_{39}}}.$$

### 4.8. Equilibrium Unfolding

We prepared 28 samples containing NTD at the concentration of 0.04 mg/mL (4.7 µM), in 5 mM sodium phosphate buffer, 50 mM NaCl and 1 mM DTT, pH 7.4, 25 °C, and urea concentrations ranging from 0.0 to 8.4 M. Fluorescence spectra were acquired in a 10 mm × 4 mm quartz cuvette, between 300 and 500 nm at 25 °C, with an excitation wavelength of 280 nm. We used a PerkinElmer LS 55 spectrofluorimeter (Waltham, MA, USA), equipped with a thermostated cell holder attached to a Haake F8 water bath (Karlsruhe, Germany). We then normalized the fluorescence spectra to the signal at 340 nm. For each spectrum, we calculated the COM, according to $\mathrm{COM}={\left[\left(\underset{i}{\sum }\nu_{i}\cdot F_{i}\right)/\left(\underset{i}{\sum }F_{i}\right)\right]}^{-1}$, where $F_{i}$ is the fluorescence emission at a wavenumber of $\nu_{i}$. We plotted the resulting COM values as a function of urea concentration and fitted the traces obtained to the equation of a two-state transition model, edited by Santoro and Bolen [37]. The output of this analysis yielded the free energy change of unfolding in the absence of denaturant $\Delta G_{U-F}^{H_{2}O}$ and the dependence of free energy change on urea concentration ($m_{\mathrm{eq}})$. We then calculated the urea concentration of middle denaturation ($C_{m})$, according to $C_{m}=\Delta G_{U-F}^{H_{2}O}/m_{\mathrm{eq}}$. We employed the same protocol to study the equilibrium denaturation of fluorescently labeled mutants (see below). The only differences were a different window of fluorescence emission (300–450 nm), a 3 mm × 3 mm quartz cuvette and a Cary Eclipse Fluorescence Spectrophotometer (Agilent Technologies, Santa Clara, California, USA), equipped with a thermostated cell holder attached to a PCB 1500 water Peltier system (Agilent technologies).

We also studied the urea-induced denaturation of NTD by means of far-UV CD, using the band at 233 nm. We prepared 24 samples containing 0.2 mg/mL (23.5 µM) NTD in 5 mM sodium phosphate buffer, 50 mM NaCl and 1 mM DTT, pH 7.4, 25 °C. Far-UV CD spectra were recorded at 25 °C using a 1 mm quartz cuvette in a JASCO J-810 spectropolarimeter (Tokyo, Japan) equipped with a thermostated cell holder attached to a Thermo Haake C25P water bath (Karlsruhe, Germany). Data were blank subtracted, masked when the HT signal was higher than 600 V and normalized to mean residue ellipticity (Θ; deg cm^2^ dmol^−1^). The resulting values of Θ at 233 nm were plotted vs. urea concentration and fitted with the equation of a two-state transition model, described by Santoro and Bolen [37].

### 4.9. Differential Scanning Fluorimetry

The fractions from analytical gel filtration containing the NTD dimeric protein were subjected to thermal denaturation through the DSF technique, using the CFX96 Touch Deep Well Real-time PCR (Bio-Rad, Hercules, CA, USA). NTD at a final concentration of 0.25 mg/mL (29 µM), was mixed with 0.5 μL of Sypro Orange dye in dimethyl sulfoxide (DMSO, ThermoFisher Scientific, Waltham, MA, USA), in 5 mM sodium phosphate buffer and 50 mM NaCl, pH 7.4, to a final volume of 50 μL. For each measurement, 20 μL of this mixture were added into a low-profile hard-shell 96-well PCR plate (#1725270), sealed with PX1 PCR Plate Sealer (#1814000) (all from Bio-Rad). All samples were prepared in duplicate. The samples were heated with a ramp speed (2 min at 25 °C, followed by a ramp to 95 °C over 90 min). The Sypro Orange fluorescence was plotted vs. temperature. In order to obtain the melting temperature ($T_{m}$), we fitted experimental curves to an adapted form of a Boltzmann distribution [53].

### 4.10. Acid-Induced Denaturation

Thirty-three samples containing 0.02 mg/mL (2.65 μM) NTD were prepared in solutions containing a 10 mM concentration of a given buffer at different pH values, with a total ionic strength of 30 mM (using NaCl). We employed citrate (pH 2.2-2.8), formate (pH 2.9–3.9), acetate (pH 3.9–5.6), MES (pH 5.6–6.5), phosphate (6.5–7.5), Tris (pH 7.5–8.8) and borate (pH 8.8–10.0). All these buffers were purchased from Sigma-Aldrich. Of the samples 200 μL were incubated for 1 h at 25 °C, and the fluorescence spectra were acquired between 300 and 450 nm, using an excitation wavelength of 280 nm, with a PerkinElmer LS-55 spectrofluorometer (Waltham, MA) and a 10 mm × 2 mm quartz cuvette. We calculated the COM for each spectrum, as described above.

### 4.11. Unfolding/Refolding Kinetics

Unfolding/refolding kinetics of NTD TDP-43 were followed in real time using a Bio-Logic (Claix, France) SFM-3 stopped-flow device, equipped with a FC-08 cuvette, coupled to a fluorescence detection system and thermostated with a Haake F8 water bath (Karlsruhe, Germany). All the experiments were performed in 5 mM sodium phosphate buffer, 50 mM NaCl and 1 mM DTT, pH 7.4, 25 °C, with a final NTD concentration of 0.03 mg/mL (3.5 µM), using an excitation wavelength of 280 nm and a band-pass filter for cutting emitted fluorescence below 320 nm. The dead time was generally 6.1 ms. For the refolding experiments, a sample containing NTD unfolded in 5.5 M urea was diluted 10-fold into refolding buffers containing lower urea concentrations, to final values ranging from 0.55 to 2.85 M. In another experiment, the unfolded protein was diluted 20-fold, to reach a final urea and protein concentrations of 0.275 M and 1.75 µM, respectively. The refolding traces were fitted to a double exponential equation of the type $F=a\cdot t+b+A_{1}\cdot \exp\left(-k_{1}\cdot t\right)+A_{2}\cdot \exp\left(-k_{2}\cdot t\right)$, where $A_{1}$ and $A_{2}$ are the amplitudes of the two refolding phases, $k_{1}$ and $k_{2}$ are their rate constants, a and b are the coefficients that account for the linear dependence of the plateau signal on time t. For the unfolding experiments, a sample containing folded NTD in 2.0 M urea was diluted 10-fold into denaturing buffers containing increasing concentrations of urea, with final concentrations ranging from 4.2 to 7.7 M. The traces obtained were fitted to a single exponential equation of the type $F=a\cdot t+b+A\cdot \exp\left(-k_{u}\cdot t\right)$, where A and $k_{u}$ are the amplitude and rate constant of the unfolding phase, respectively. We also followed NTD refolding using the PerkinElmer LS 55 spectrofluorimeter mentioned above. Dead time was generally 15 s. We acquired fluorescence at 350 nm, using an excitation wavelength of 280 nm and excitation/emission slits of 5 nm. The traces obtained were fitted to a double exponential equation of the type $F=a\cdot t+b+A_{3}\cdot \exp\left(-k_{3}\cdot t\right)+A_{4}\cdot \exp\left(-k_{4}\cdot t\right)$, where, again, $A_{3}$ and $A_{4}$ are the amplitudes of the two observed phases, $k_{3}$ and $k_{4}$ are their rate constants. The values of the kinetic constants $k_{1}$, $k_{2}$, $k_{3}$, $k_{4}$ and $k_{u}$ were plotted vs. the denaturant concentration to obtain the so-called chevron plot. We analysed the chevron plot of $k_{2}$ and $k_{u}$ in two ways. First, the plot was fitted to the two-state model edited by Jackson and Fersht [54]:

(3) $$k_{obs}=k_{2}^{H_{2}O}\cdot \exp\left\{-m_{2}/\left(RT\right)\cdot \left[\mathrm{urea}\right]\right\}+k_{u}^{H_{2}O}\cdot \exp\left\{m_{u}/\left(RT\right)\cdot \left[\mathrm{urea}\right]\right\}$$

where $k_{2}^{H_{2}O}$ and $k_{u}^{H_{2}O}$ are the folding and unfolding constants in the absence of the denaturant, *m*_2_ and *m*_u_ indicate the dependence of $k_{2}$ and $k_{u}$ on urea concentration, R is the ideal gas constant and T is the temperature. We used the fit output values obtained with Equation (3) to reproduce independently the equilibrium values. Indeed, $\Delta G_{U-F}^{H_{2}O}=-R\cdot T\cdot \ln\left(k_{u}^{H_{2}O}/k_{2}^{H_{2}O}\right)$, $m_{\mathrm{eq}}=m_{2}+m_{u}$ and $C_{m}=R\cdot T/\left(m_{2}+m_{u}\right)\cdot \ln\left(k_{2}^{H_{2}O}/k_{u}^{H_{2}O}\right)$. Second, to check the compatibility between equilibrium and kinetic data, we modified Equation (3) to impose the $\Delta G_{U-F}^{H_{2}O}$ and $m_{\mathrm{eq}}$ obtained from equilibrium experiments:

(4) $$k_{obs}=k_{2}^{H_{2}O}\cdot \exp\left\{-m_{2}/\left(RT\right)\cdot \left[\mathrm{urea}\right]\right\}+\left\{k_{2}^{H_{2}O}\cdot \exp\left[-\Delta G_{U-F}^{H_{2}O}/\left(RT\right)\right]\right\}\cdot \exp\left\{\left(m_{\mathrm{eq}}-m_{f}\right)/\left(RT\right)\cdot \left[\mathrm{urea}\right]\right\}$$

We repeated the experiments using final NTD concentrations ranging from 0.01 to 0.1 mg/mL (1.16–11.6 µM), at a final urea concentration of 1.6 M. In a last experiment, we monitored in real-time NTD refolding at 0.55 M urea and 0.03 mg/mL (3.5 µM) protein, by a 10-fold dilution of a solution containing 0.3 mg/mL NTD in 5.5 M urea. We then compared the fluorescence emissions observed at the beginning of the refolding trace (t = 0 µs) with that of the unfolded protein under the same conditions, extrapolated linearly from measurements of unfolded NTD at high urea concentrations (4.0 and 8.0 M urea).

### 4.12. Dimerization Kinetics Using FRET

We prepared two samples containing 39D-39A or 50D-50A (1:1 molar ratio), at a total protein concentration of 0.25 mg /mL (29 µM), in 4.5 M urea, 5 mM sodium phosphate buffer, 50 mM NaCl, pH 7.4 and 1 mM DTT. Refolding was induced by a 10-fold dilution of such solutions into a refolding buffer at 25 °C. We monitored the kinetics of dimerization upon refolding for 60 s, using the SFM-3 stopped-flow device equipped with a FC-08 cuvette, an excitation wavelength of 336 nm and a band-pass filter to cut emitted fluorescence below 470 nm. Data were fitted to an exponential equation of the form $F=a\cdot t+b+A_{1}\cdot \exp\left(-k_{1}\cdot t\right)+A_{2}\cdot \exp\left(-k_{2}\cdot t\right)$.

### 4.13. Proline Isomerization

We dissolved NTD at 0.3 mg/mL (35 µM) in 5 mM sodium phosphate buffer and 50 mM NaCl, pH 7.4 with 5.0 M urea for 30 min at 25 °C. After this time, we prepared four solutions containing different concentrations of the macrophage infectivity potentiator (Mip) protein (kindly gifted by Prof. Nicholas J. Harmer, University of Exeter, UK), known to possess PPI activity, in 5 mM phosphate buffer and 50 mM NaCl, pH 7.4. For each of the four solutions, folding was initiated by adding 60 µL of the NTD-containing solution to 540 µL of the Mip-containing solution in cuvette. Final conditions were 0.03 mg/mL (3.5 µM) NTD, 0, 112, 225 or 450 nM Mip, 0.5 M urea, 5 mM sodium phosphate buffer, 50 mM NaCl, pH 7.4, 25 °C. Fluorescence was recorded for at least 3600 s in a 10 mm × 2 mm quartz cuvette at 25 °C, using excitation and emission wavelengths of 280 and 360 nm, excitation and emission slits of 2.5 and 10 nm, respectively. We used a Cary Eclipse Fluorescence Spectrophotometer (Agilent Technologies), equipped with a thermostated cell holder attached to a PCB 1500 water Peltier system (Agilent technologies).

## Supplementary Materials

Supplementary materials can be found online and contain Supplementary Methods, Figure S1, Figure S2, Figure S3, Figure S4, Figure S5, Figure S6, Figure S7, Table S1, Table S2, Table S3 and Supplementary References [10,11,12,13,35,53,55] https://www.mdpi.com/1422-0067/21/17/6259/s1.

## Abbreviations

1,5-IAEDANS	5-((((2-iodoacetyl)amino)ethyl)amino)naphthalene-1-sulfonic acid
6-IAF	6-iodoacetamidofluorescein
AD	Alzheimer’s Disease
ALS	Amyotrophic Lateral Sclerosis
β2m	β2-microglobulin
CD	Circular Dichroism
COM	Center Of spectral Mass
CS	Collapsed State
CTD	C-terminal Domain
DLS	Dynamic Light Scattering
DMF	Dimethylformamide
DMSO	Dimethyl sulfoxide
DSF	Differential Scanning Fluorimetry
DTT	Dithiothreitol
fALS	Familial Amyotrophic Lateral Sclerosis
F	Folded State
F*	Native-like State
FRET	Förster Resonance Energy Transfer
FTLD-U	Ubiquitin-positive FrontoTemporal Lobar Degeneration
HD	Huntington’s Disease
HT	High Tension
I	Intermediate State
MES	2-(N-morpholino)ethanesulfonic acid
NES	Nuclear Export Signal
NLS	Nuclear Localization Signal
NMR	Nuclear Magnetic Resonance
NOE	Nuclear Overhauser Effect
NTD	N-terminal Domain
PCR	Polymerase Chain Reaction
PD	Parkinson’s Disease
PDB	Protein Data Bank
PF	Partially Folded State
PPI	Peptidyl Prolyl Isomerase
RRM1	RNA Recognition Motif 1
RRM2	RNA Recognition Motif 2
SH3	Src Homology 3
TDP-43	TAR DNA-binding Protein 43
U	Unfolded State
