# Supplementary material for: Insight into the Folding and Dimerization Mechanisms of the N-Terminal Domain from Human TDP-43
Source: Int J Mol Sci. 2020 Aug 29;21(17):6259. doi: 10.3390/ijms21176259 (PMC7504384; doi:10.3390/ijms21176259)
Supplement: Supplementary file 1 [file ijms-21-06259-s001.pdf]

Supplementary Material to

Insight into the folding and dimerization mechanisms of the N-terminal domain from human TDP-43

Mirella Vivoli Vega [1,2], Prandvera Guri [1], Fabrizio Chiti [1] and Francesco Bemporad [1,*]

[1] Department of Experimental and Clinical Biomedical Sciences "Mario Serio", Section of Biochemistry, University of Florence, Viale G. B. Morgagni 50, 50134 Florence, Italy
[2] Present affiliation: Living Systems Institute, University of Exeter, Stocker Road, Exeter, EX4 4QD, UK
* Correspondence: francesco.bemporad@unifi.it; Tel: +39-055-2751211

Supplementary Methods

Gene cloning

Using ligation-independent cloning (LIC) [1], full-length TDP-43 was cloned from pGEX-2T plasmid into pNIC28-Bsa4 plasmid (a gift from Prof. Nicholas J. Harmer, University of Exeter, Exeter, UK) by PCR. The following primers, synthesized by Eurofins Genomics (Ebersberg, Germany), were used to amplify the TDP-43 insert from the pGEX-2T plasmid, each containing about 19 bp of sequence homology to the ends and generating single-stranded DNA overhangs: Fw: 5' –CCG AGA ACC TGT ACT TCC AAT CCA TGT CTG AAT ATA TTC GGG-3'; Rv: 5'-GGC ATT TTC TTT TGC GTT TCT ACA TTC CCC AGC CAG-3'. To produce overhangs in pNic28-Bsa4 we used the following primers: Fw: 5'-CCC GAA TAT ATT CAG ACA TGG ATT GGA AGT ACA GGT TCT CGG-3'; Rv: 5'-CTG GCT GGG GAA TGT AGA AAC GCA AAA GAA AAT GCC-3'. PCR was carried out using Q5® hot start high-fidelity DNA polymerase (New England Biolabs, Ipswich, MA) in 100 μL reaction volume. The PCR products were cleaned with the NucleoSpin Gel and PCR Clean-Up Kit (Macherey-Nagel, Düren, Germany), quantified with NanoDrop® ND-1000 spectrophotometer (Thermo Fisher Scientific, Waltham, MA) and then ligated in presence of 0.25 μL T4 DNA polymerase (Fermentas, Burlington, Canada) in 10 μL reaction volume, with 200 ng of pNic28-Bsa4 vector and 85 ng and/or 170 ng of TDP-43 insert, in a 1:2 and 1:4 molar ratio, respectively, for 2 min at room temperature. The mixture was then placed on ice for 10 min. 2 μL of LIC reaction were used to transform 50 μL of *E. coli* XL1 competent cells. Colonies from agar plates, supplemented with 50 μg mL^{-1} kanamycin, were cultured and plasmid purification performed using the extraction kit from Thermo Fisher Scientific. Transformants were characterized by colony PCR screening using the primers for insert. Detection of a single band of the expected size at ~1242 bp for His-TDP-43 by agarose gel electrophoresis was used to selected transformants. The correct nucleotide sequence was verified by DNA sequencing (BMR Genomics, Padua, Italy) and expressed the following protein sequence: a His-tag (6xHis) at the N-terminus, followed by a Tobacco Etch Virus (TEV) protease cleavage site and human TDP-43.

The resulting plasmid pNic28-Bsa4 coding for His-TDP-43 was used to carry out mutagenesis to insert a stop codon at position 78, using Quick Change II XL Site-directed Mutagenesis kit (Agilent Technologies, Santa Clara, CA, USA). The following primers were used to perform mutagenesis with PCR: Fw: 5'- GGT GTA TGT TGT CAA CTA TTG AAA AGA TAA CAA AAG AAA AAT GG-3'; Rv: 5'- CCA TTT TTC TTT TGT TAT CTT TTC AAT AGT TGA CAA CAT ACA CC-3'. The presence of the stop codon was confirmed by DNA sequencing (BMR Genomics, Padua, Italy). The resulting plasmid pNic28-Bsa4, coding for 6xHis, followed by TEV protease cleavage site and the first 77 residues of TDP-43 (hereinafter referred to as NTD) was transformed into *E. coli* BL21 (DE3) cells (New England Biolabs, Ipswich, Massachusetts), which were grown in 20 g/L autoclaved LB medium (Sigma Aldrich, St. Louis, MI, USA), supplemented with 50 μg mL^{-1} kanamycin.

Mutagenesis

Quick Change II XL Site-directed Mutagenesis kit (Agilent Technologies) was used to obtain separately the single-point mutations Cys39Ser (C39S) and Cys50Ser (C50S), with the aim of obtaining two mutants containing only one cysteine residue instead of two. The following primers contained the codon to replace the cysteine residue with serine. We used the following primers the mutation C39S: Fw: 5′- GGT TTC CAG GGG CGA GTG GGC TTC GCT A-3′; Rv: 5′- TAG CGA AGC CCA CTC GCC CCT GGA AAC-3′

We used the following primers the mutation C50S: Fw: 5′- GGA ATC CAG TGT CTC AGA GTA TGA GAG GTG TCC GGC-3′; Rv: 5′- GCC GGA CAC CTC TCA TAC TCT GAG ACA CTG GAT TCC-3′.

The presence of the missense codon was confirmed by DNA sequencing (BMR genomics, Padua, Italy). The resulting plasmids were transformed into E. coli BL21 (DE3) cells (New England Biolabs), similarly to the plasmid containing the wild-type sequence.

Protein Expression

4 L of LB medium (Sigma Aldrich, St. Louis, MI, USA) were prepared (20 g/L), autoclaved and supplemented with 50 μg mL^{-1} kanamycin. A 100 mL pre-culture of the same LB medium containing the *E. coli* BL21 (DE) cells previously transformed with the pNic28-Bsa4 plasmid (as described above) was incubated at 37 °C for about 20 h. The next day, 20 mL of the pre-culture were transferred to 1 L of LB medium (this was repeated for all the 4 L of medium). After about 3 h of growth at 37 °C and OD_{600} = 0.6 the bacterial cell cultures were induced with 0.5 mM isopropil-β-D-1-tiogalattopiranoside (IPTG) and grown at 25 °C for at least 22 h. The bacteria were then harvested at 5000 g for 30 min at 4 °C. The bacterial pellets were resuspended in 10 mL (for each centrifuge tube) of Buffer A (50 mM Tris-HCl, pH 8.0, 0.5 M NaCl, 10 mM Imidazole) and cell lysis was performed adding 1 mL of 50 mg/mL lysozyme and 1 tablet of Pierce protease inhibitors EDTA free (Thermo Fischer Scientific). After 1 h incubation at room temperature, the bacterial solutions were sonicated on ice at 70 kHz (6 cycles of 20 s each) and the lysate centrifuged at 24000 g for 30 min, at 4 °C, to separate the pellet (containing the cell debris) from the supernatant (containing the soluble protein).

Protein Purification

A 5 mL HisTrap FF (GE Healthcare, IL, USA) column was pre-equilibrated at 4 °C using buffer A. The protein-containing supernatant was loaded at 4 °C onto the column using a FPLC Akta pure 25 L system (GE Healthcare, IL, USA) at a flow rate of 3 ml/min. A wash step was performed using 10 column volumes of 25% of buffer B (50 mM Tris-HCl, pH 8.0, 0.5 M NaCl, 250 mM imidazole) and the protein eluted with 10 column volumes of 50% buffer B. Fractions containing the protein were pooled and concentrated down to 5 ml at 4 °C by ultrafiltration at 3 bar, using a 50 mL Amicon stirred cell with a 3 kDa cut-off cellulose membrane (Millipore, MA, USA), or, alternatively, by centrifugation at 5000 g, using a Pierce® 3 kDa cut-off polyethersulfone (PES) concentrator (Thermo Fischer Scientific). The resulting 5 mL sample were loaded onto the HiLoad 16/600 Superdex 200 pg column using a 5 mL capillary loop and isocratically eluted at 1 ml/min with 5 mM Na_2HPO_4, pH 7.4, 150 mM NaCl, at 4 °C. The main peak fractions were pooled and the concentration calculated using a SHIMADZU UV-1900 uv-vis spectrophotometer at a wavelength of 280 nm, with an extinction coefficient of 12950 $M^{-1}cm^{-1}$ (assuming all Cys residues are reduced) and a molecular weight of 11081.29 Da.

The pooled fractions were treated with a His-tagged TEV protease, in a ratio of 1:4 (TEV:protein), in the presence of 10% glycerol, in 50 mM Tris-HCl, pH 8.5, 1 mM EDTA, 1 mM DTT, under gentle stirring for at least 15 h at 18 °C. The sample was then loaded, at 4 °C, on a 10 mL HisPur™ Ni-NTA resin (Thermo Fisher Scientific) in a gravity flow column pre-equilibrated with buffer A. The flow-through, containing the protein devoid of the His-tagged TEV, was collected and concentrated down to 5 ml by centrifugation at 5000 g, 4 °C, using the Pierce® 3 kDa cut-off protein PES concentrator, followed by a buffer exchange using 5 mM Na_2HPO_4,

50 mM NaCl, 1 mM DTT, pH 7.4, under the same conditions. The concentration of protein sample was calculated using an extinction coefficient of 11460 $M^{-1}cm^{-1}$ (assuming all Cys residues are reduced) and a molecular weight of 8615.64 Da. Protein samples were analyzed electrophoretically, by SDS-PAGE, using 4–20% Mini-Protean TGX Precast polyacrylamide gels (Bio-Rad, Hercules, CA, USA) and chromatographically, by gel filtration with HiLoad 16/600 Superdex 200 pg. The final NTD sample was electrophoretically and chromatographically pure (> 95%). It contained 77 residues and an additional serine residue at the N-terminus resulting from the TEV cleavage site (numbered Ser0).

Supplementary figures and tables

Figure S1

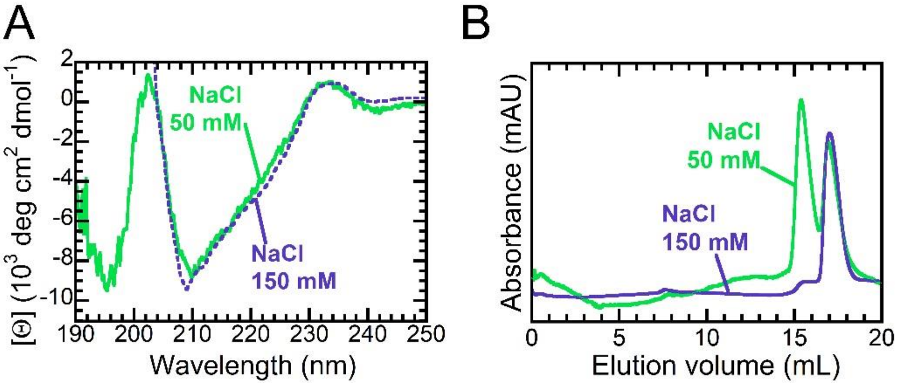

Figure S1: Secondary structure and oligomeric state of NTD in the presence of 150 mM NaCl. (A) Comparison between the far-UV CD spectra of NTD in the presence of 50 mM NaCl (green continuous line) and 150 mM NaCl (purple dashed line). The spectra were cut when the HT signal was higher than 600 V. (B) Analytical gel filtration of NTD in the presence of 50 mM NaCl (green line) or 150 mM NaCl (purple line). The peaks observed are placed at 15.38 and 16.95 ml in the presence of 50 mM NaCl, at 15.54 and 17.02 ml in the presence of 150 mM NaCl.

Figure S2

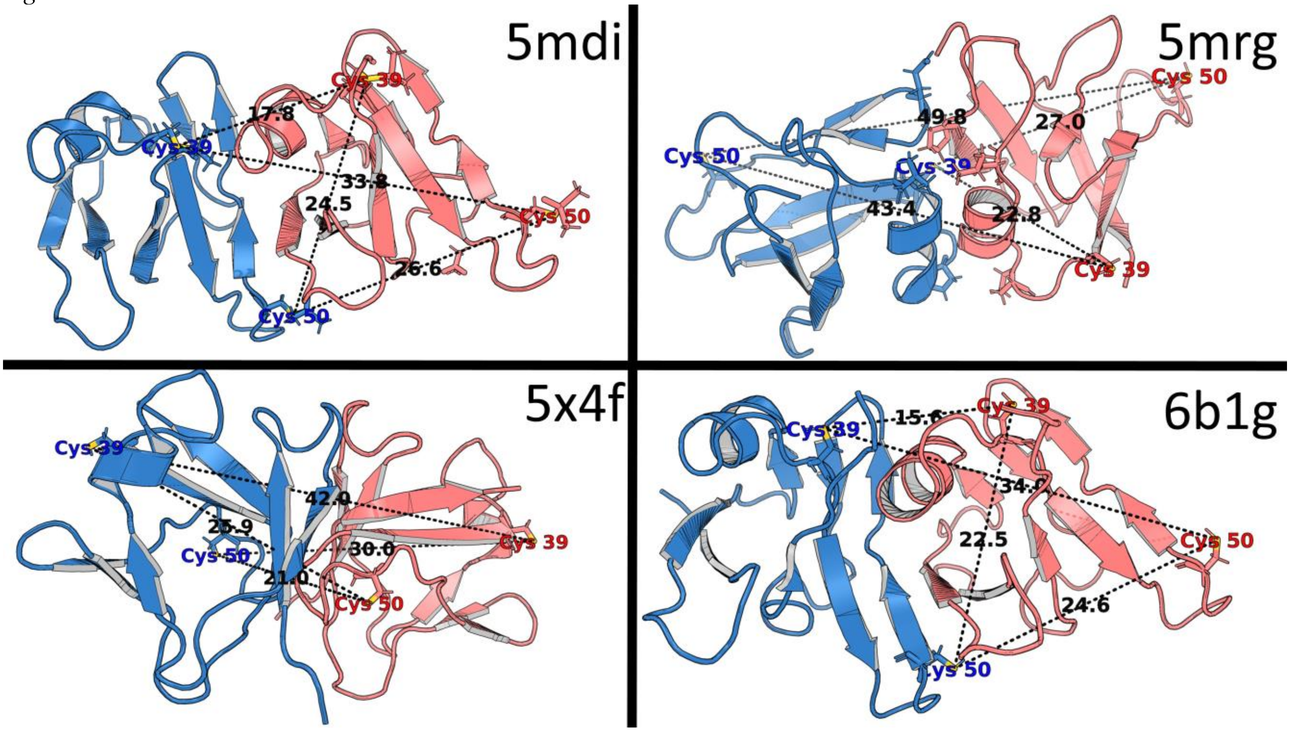

Figure S2: Structures or structural models of NTD dimers proposed by different authors: PDB entry 5mdi [2], 5mrg [3], 5x4f [4], 6b1g [5]. Cyan and pink colors correspond to the two different monomers. The 5mrg and 5x4f PDB entries only describe the structure of a monomer and the dimer is imaged in this figure based on the information provided by the authors on the monomer-to-monomer interface. Black numbers indicate spatial distances between the indicated cysteine residues in Å. The distances are summarized in Table S2.

Figure S3

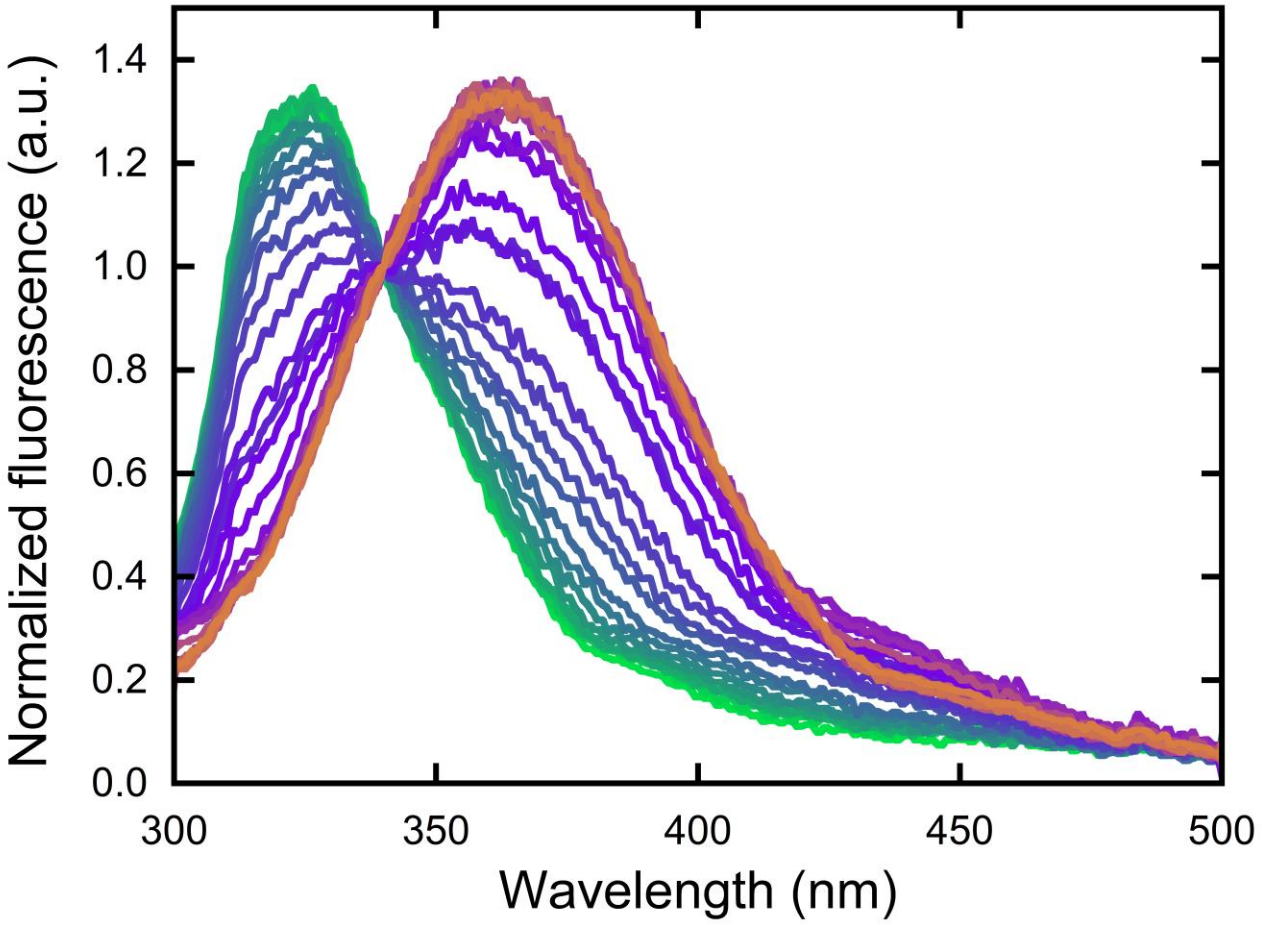

Figure S3: Tryptophan fluorescence spectra of NTD measured at different urea concentrations, ranging from 0 (green) to 8.2 (orange) M urea.

Figure S4

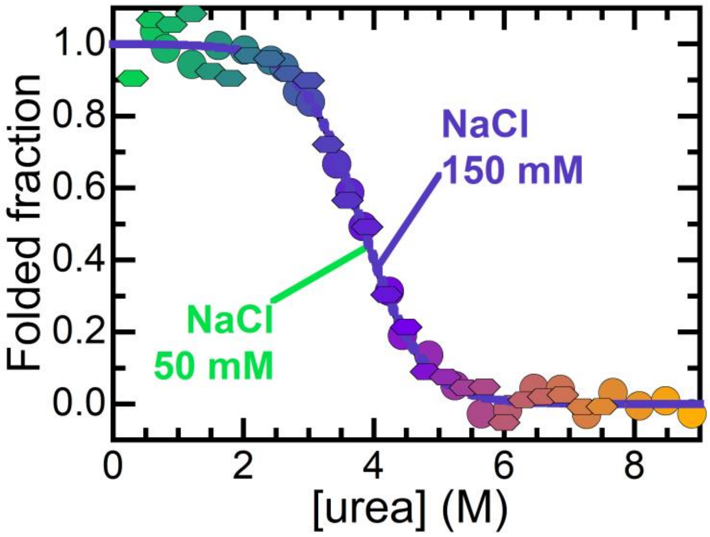

Figure S4: Comparison between the urea induced denaturation curves of NTD carried out in the presence of 50 mM NaCl (circles) and 150 mM NaCl (hexagons). Continuous lines represents best fits of experimentaltraces to a two-state model. In the presence of 50 mM NaCl, this analysis yielded values of 20.1 ± 1.5 kJ/mol, 5.3 ± 0.3 kJ/(mol M) and 3.81 ± 0.20 M for $\Delta G_{U-F}^{\mathrm{H_2O}}$, m_{eq} and C_{m}, respectively. In the presence of 150 mM NaCl we obtained values of 19.5 ± 1.5 kJ/mol, 5.1 ± 0.3 kJ/(mol M) and 3.82 ± 0.20 M for $\Delta G_{U-F}^{\mathrm{H_2O}}$, m_{eq} and C_{m}, respectively.

Figure S5

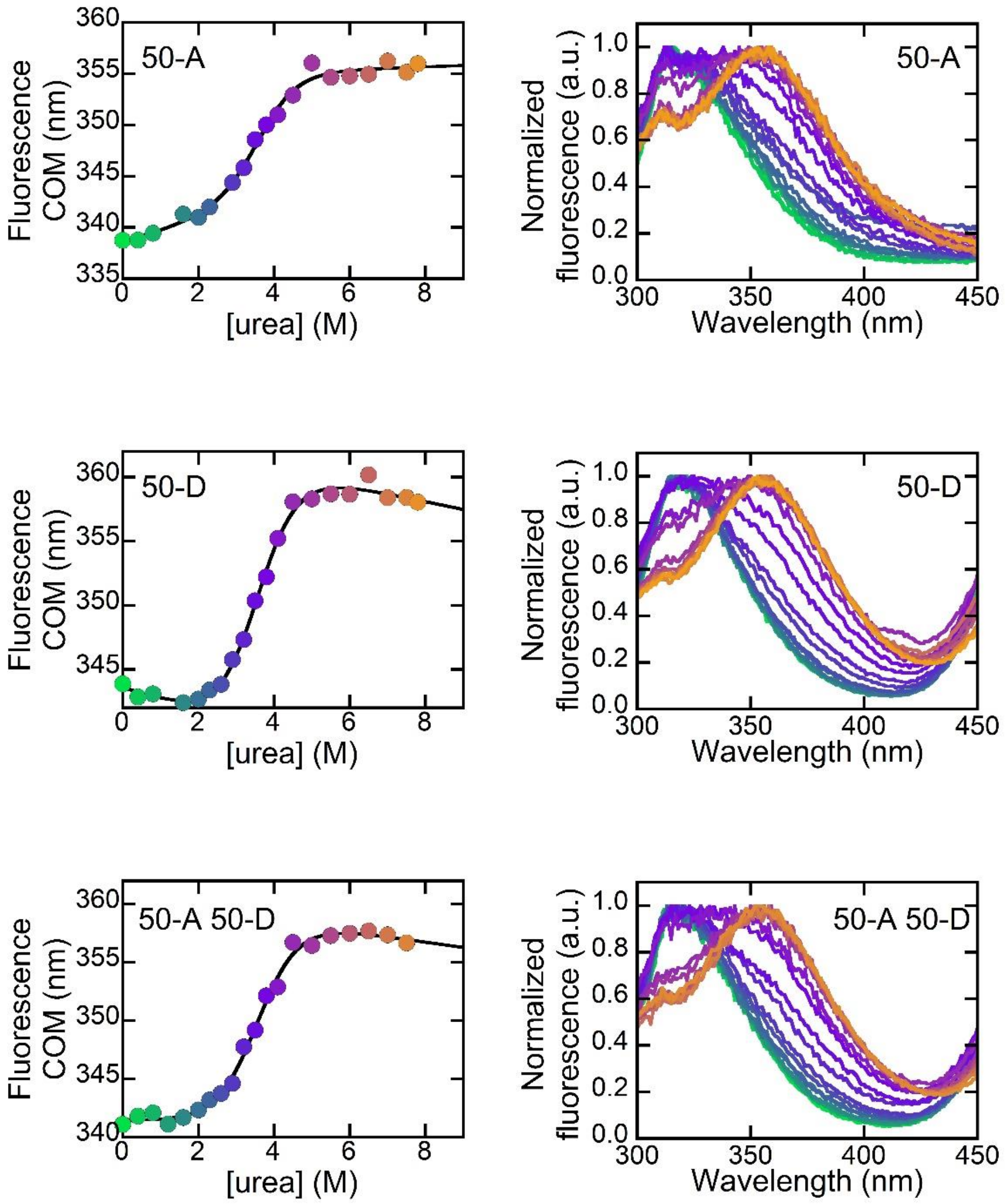

Figure S5: Urea induced equilibrium denaturation of C39S NTD labelled at position 50 with either the acceptor (50-A, top row), the donor (50-D, middle row), or a 50:50 molar ratio of the two labels (50-A 50-D, bottom row). Left-hand panels report the fluorescence centre of mass (COM) as a function of urea concentration. We calculated COM values from the corresponding spectra shown in the right-hand panels. Fluorescence spectra were normalized to the maximum. In all panels colour ranges from green to orange upon urea concentrations increasing from 0 to 7.8 M.

Figure S6

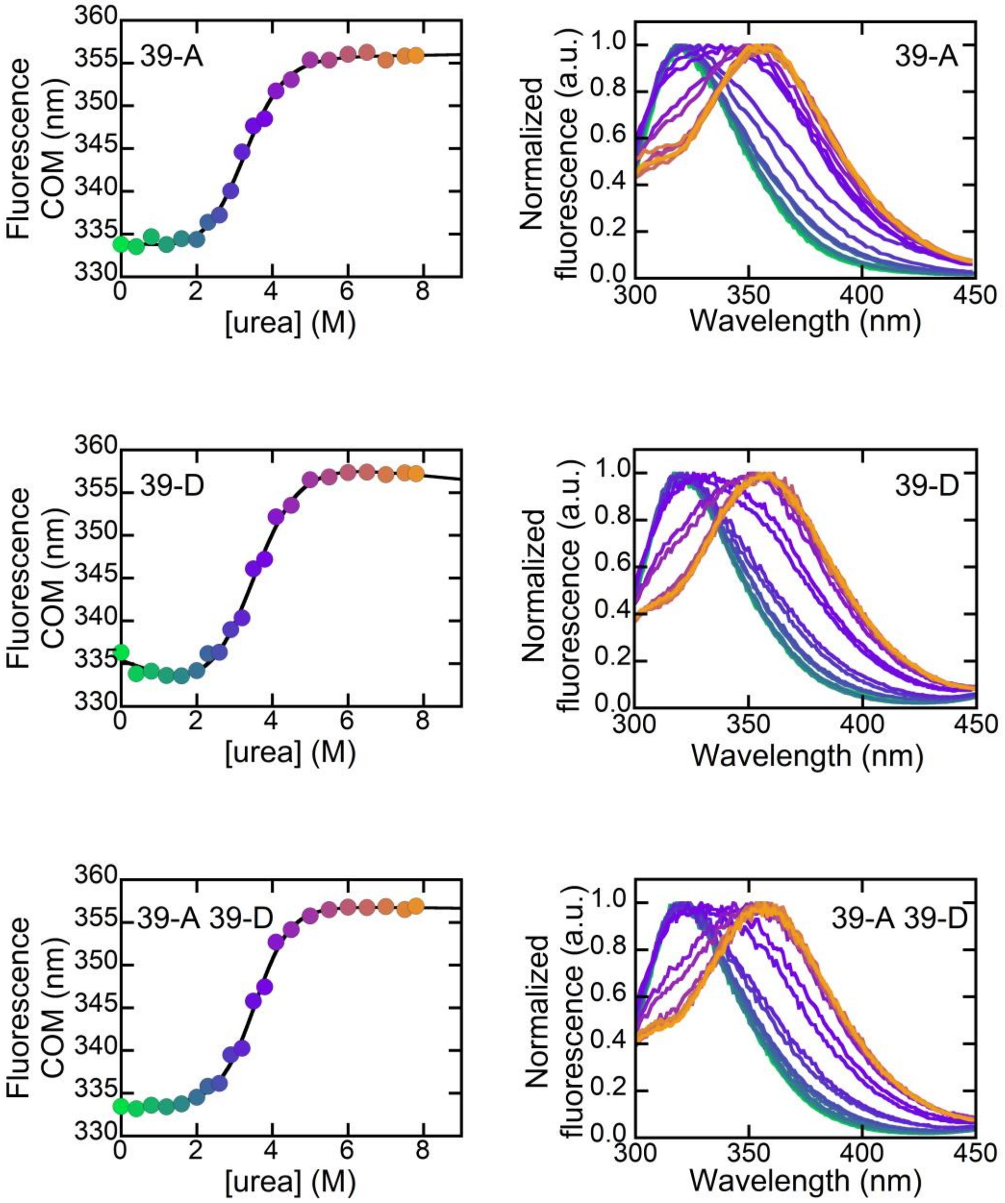

Figure S6: Urea induced equilibrium denaturation of C50S NTD labelled at position 39 with either the acceptor (39-A, top row), the donor (39-D, middle row), or a 50:50 molar ratio of the two labels (39-A 39-D, bottom row). Left-hand panels report the fluorescence centre of mass (COM) as a function of urea concentration. We calculated COM values from the corresponding spectra shown in the right-hand panels. Fluorescence spectra were normalized to the maximum. In all panels colour ranges from green to orange upon urea concentrations increasing from 0 to 7.8 M.

Figure S7

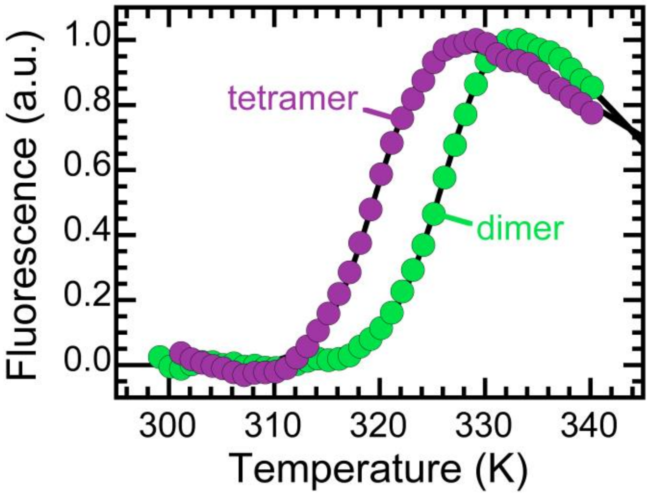

Figure S7: Comparison between thermal unfolding of dimeric (green) and tetrameric (purple) NTD, carried out under identical conditions. Protein concentration was 0.25 mg/ml. Continuous lines represent best fit to an adapted Boltzmann distribution [6]. T_m values are 52.8 °C (326 K) and 47.2 °C (320.4 K) for dimer and tetramer, respectively.

Table S1

<table>
<tr><th>Secondary structure type</th><th>%</th><th>Substructure</th><th>%</th></tr>
<tr><td rowspan="2">Helix</td><td rowspan="2">12.6</td><td>Helix 1 (regular)</td><td>8.8</td></tr>
<tr><td>Helix 2 (distorted)</td><td>3.8</td></tr>
<tr><td rowspan="3">Antiparallel</td><td rowspan="3">40.4</td><td>Anti1 (left-twisted)</td><td>2.7</td></tr>
<tr><td>Anti2 (relaxed)</td><td>10.5</td></tr>
<tr><td>Anti3 (right-twisted)</td><td>27.2</td></tr>
<tr><td>Parallel</td><td>1.7</td><td colspan="2" rowspan="3"></td></tr>
<tr><td>Turn</td><td>9.6</td></tr>
<tr><td>Other</td><td>35.7</td></tr>
</table>

Table S1: Output of the deconvolution with BeStSel [7] of the far-UV CD spectrum for the native protein in the absence of urea reported in Figure 2A in the main text.

Table S2

PDB entry	references	r_{50-50} (Å)	r_{39-50} (Å)	r_{50-39} (Å)	r_{39-39} (Å)	r_{39-39}/r_{50-50}
**5mdi**	[2]	26.6	33.8	24.5	17.8	0.67
**5mrg**	[3]	49.8	43.4	27.0	22.8	0.46
**5x4f**	[4]	21.0	30.0	25.9	42.0	2.00
**6b1g**	[5]	24.6	34.0	22.5	15.6	0.63

Table S2: Intermolecular spatial distances among Cys39 and Cys50 residues in the NTD homodimers corresponding to the PDB entries listed in the first column. Distances are reported in Å and were obtained from Figure S2.

Table S3

**Variant**	$\Delta G_{U-F}^{\mathrm{H_2O}} \left(\frac{\mathrm{KJ}}{\mathrm{mol}}\right)$	$m_{\mathrm{eq}} \left(\frac{\mathrm{KJ}}{\mathrm{mol\ M}}\right)$	$C_{\mathrm{m}}(\mathrm{M})$
**WT (Fluorescence)**	20.1 ± 1.5	5.3 ± 0.3	3.81 ± 0.20
**WT (CD)**	21.8 ± 1.5	5.3 ± 0.3	4.13 ± 0.20
**39A**	14.9 ± 1.5	4.6 ± 0.3	3.22 ± 0.20
**39D**	13.9 ± 1.5	4.1 ± 0.3	3.42 ± 0.20
**39D-39A**	17.0 ± 1.5	4.7 ± 0.3	3.55 ± 0.20
**50A**	17.3 ± 1.5	4.7 ± 0.3	3.63 ± 0.20
**50D**	16.8 ± 1.5	4.7 ± 0.3	3.55 ± 0.20
**50D-50A**	16.5 ± 1.5	4.6 ± 0.3	3.58 ± 0.20

Table S3: Thermodynamic parameters for the equilibrium unfolding experiments carried out on different NTD variants under the same conditions. We analyzed data as reported in the Methods section, in the main text.

Supplementary references

1. Li, M.Z.; Elledge, S.J. Harnessing homologous recombination in vitro to generate recombinant DNA via SLIC. *Nat Meth* **2007**, *4*, 251-6.
2. Afroz, T.; Hock, E.M.; Ernst, P.; Foglieni, C.; Jambeau, M.; Gilhespy, L.A.B.; Laferriere, F.; Maniecka, Z.; Pluckthun, A.; Mittl, P., et al. Functional and dynamic polymerization of the ALS-linked protein TDP-43 antagonizes its pathologic aggregation. *Nat Comm* **2017**, *8*, 45.
3. Mompean, M.; Romano, V.; Pantoja-Uceda, D.; Stuani, C.; Baralle, F.E.; Buratti, E.; Laurents, D.V. Point mutations in the N-terminal domain of transactive response DNA-binding protein 43 kDa (TDP-43) compromise its stability, dimerization, and functions. *J Biol Chem* **2017**, *292*, 11992-12006.
4. Jiang, L.L.; Xue, W.; Hong, J.Y.; Zhang, J.T.; Li, M.J.; Yu, S.N.; He, J.H.; Hu, H.Y. The N-terminal dimerization is required for TDP-43 splicing activity. *Sci Rep* **2017**, *7*, 6196.
5. Wang, A.; Conicella, A.E.; Schmidt, H.B.; Martin, E.W.; Rhoads, S.N.; Reeb, A.N.; Nourse, A.; Ramirez Montero, D.; Ryan, V.H.; Rohatgi, R., et al. A single N-terminal phosphomimic disrupts TDP-43 polymerization, phase separation, and RNA splicing. *EMBO J* **2018**, *37*.
6. Szyperski, T.; Mills, J.L.; Perl, D.; Balbach, J. Combined NMR-observation of cold denaturation in supercooled water and heat denaturation enables accurate measurement of deltaC(p) of protein unfolding. *Eur Biophys J* **2006**, *35*, 363-6.
7. Micsonai, A.; Wien, F.; Kernya, L.; Lee, Y.H.; Goto, Y.; Refregiers, M.; Kardos, J. Accurate secondary structure prediction and fold recognition for circular dichroism spectroscopy. *Proc Natl Acad Sci U S A* **2015**, *112*, E3095-103.
